# Bond Trading: Intramolecular Metal and Ligand Exchange within a NO/Ni/Co Complex

**DOI:** 10.1002/advs.202307113

**Published:** 2023-12-03

**Authors:** Manish Jana, Xueyan Zheng, Trung Le, Manuel Quiroz, Paulina Guererro‐Almaraz, Donald J. Darensbourg, Marcetta Y. Darensbourg

**Affiliations:** ^1^ Department of Chemistry Texas A&M University College Station TX 77843 USA

**Keywords:** bond trading, heterobimetallic complexes, N_2_S_2_, nitric oxide, NO transfer

## Abstract

With the goal of generating hetero‐redox levels on metals as well as on nitric oxide (NO), metallodithiolate (N_2_S_2_)Co^III^(NO^−^), N_2_S_2_ = *N*,*N*‐ dibenzyl‐3,7‐diazanonane‐1,9‐dithiolate, is introduced as ligand to a well‐characterized labile [Ni^0^(NO)^+^] synthon. The reaction between [Ni^0^(NO^+^)] and [Co^III^(NO^−^)] has led to a remarkable electronic and ligand redistribution to form a heterobimetallic dinitrosyl cobalt [(N_2_S_2_)Ni^II^∙Co(NO)_2_]^+^ complex with formal two electron oxidation state switches concomitant with the nickel extraction or transfer as Ni^II^ into the N_2_S_2_ ligand binding site. To date, this is the first reported heterobimetallic cobalt dinitrosyl complex.

## Graphical abstract

A sulfur‐bridged {Co^III^(NO^−^)}^8^/ {Ni^0^(NO)^+^}^10^ adduct, analogous to the well‐characterized (bipyridine){Ni^0^(NO)^+^}^10^, shows a remarkable redistribution of oxidation states during transformation to d^8^‐Ni^2+^/ {Co^3+^((NO)^−^)_2_}^10^, via an intra‐, bimolecular, NO‐coupled electron transfer.

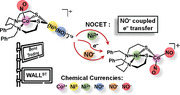

## 1 Introduction

The extreme reactivity of the nitric oxide (NO) molecule that leads to its usefulness in human physiology requires transfer between various stabilizing entities.^[^ ^1^ ^]^ Both thiols and metals serve in this capacity.^[^ ^2^ ^]^ Related to its existence in three redox levels, NO^+^, NO^∙^ and NO^−^, with π orbitals well matched to frontier orbitals of transition metals, nitric oxide (NO) is a versatile donor ligand in coordination chemistry, well known to form classes of complexes that display its multiple redox states, frequently inextricably entwined with those of the metal to which it binds.^[^ ^3^ ^]^ Such ambiguity is the genesis of the Enemark‐Feltham (E‐F) notation in transition metal nitrosyls, {M(NO)}^n^, that sums the valence d‐electrons of M with the frontier *π*‐electrons of NO, without commitment to the oxidation state of the metal or the charge on the NO.^[^ ^4^ ^]^

Synthetic programs that target heterobimetallic nitrosyl complexes bridged by sulfur atoms are of potential importance in creating and understanding redox non‐innocence in the M─NO units.^[^ ^5^ ^]^ In this regard we have explored metal nitrosyls in combination with exogeneous metals in attempts to unravel redox behaviours in M and in NO. Thus, while developing metal‐nitrosyls as synthons in the formation of isolable hetero‐bi and trimetallic complexes, we discovered an interesting “bond exchange” reaction involving Co(NO) within a N_2_S_2_ ligand field and the molybdenum(II) synthon, [Mo_2_(CH_3_CN)_10_]^4+^.^[^ ^6^ ^]^ Pursuing the concept of [(N_2_S_2_)Co(NO)] as a NO source in the formation of nitrosylated heterobimetallics, and using the interesting [Ni(NO)(CH_3_NO_2_)_3_]^+^ synthon ({Ni^0^(NO^+^)}^10^),^[^ ^7^ ^]^ we set out to explore the possibility of generating hetero‐redox levels in {Co(NO)∙Ni(NO)} bimetallics containing NO^−^ on Co^III^ and NO^+^ on Ni^0^. These bimetallic systems are of potential significance for understanding the mechanism of coupling between two NO molecules (via NO reduction) that generates the greenhouse gas N_2_O as was seen for the single metal site as well for the [(bipy)_2_Ni(NO)]^+^, bipy = bipyridine^[^ ^8^ ^]^ Another interesting aspect of the current work is to study the analogy between bidentate “bipy” and nitrosylated metallodithiolate as ligands for the {Ni(NO)}^1^ ^0^ synthon. In fact, we observed NO transfer and/or rearrangement in the S‐bridged Co─Ni bimetallic, concomitant with bond exchange and considerable electron rearrangement governed by thermodynamic preferences.

## 2 Results and Discussion

**Figure 1:**
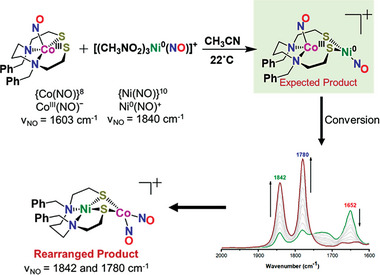
Reaction of (dadt^Bz^)Co(NO) with [Ni(NO)(CH_3_NO_2_)_3_]^+^ at 22 °C and generation of [(dadt^Bz^)Ni∙Co(NO)_2_]^+^ monitored in CH_3_CN by ν(NO) IR spectroscopy. The time course monitor is from 1 min to ca. 240 mins with disappearance of intermediate at ca. 1652 cm^−1^.

**Figure 2:**
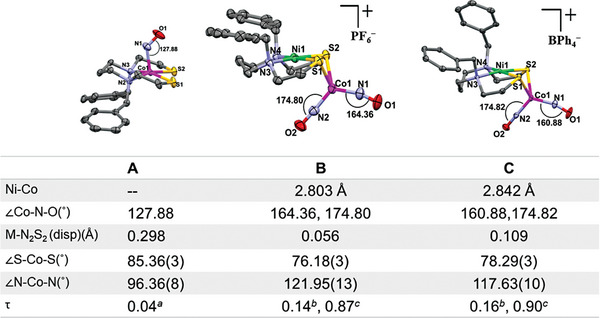
Molecular structures of (dadt^Bz^)Co(NO) (A), [(dadt^Bz^)Ni∙Co (NO)_2_][PF_6_] (B) and [(dadt^Bz^)Ni∙Co(NO)_2_][BPh_4_] (C) showing 50% probability ellipsoids with PF_6_^−^ and BPh_4_^−^ counterion omitted in the (B) and (C) structures for clarity. A full list of crystal data and structure refinements are given in Table S2 (Supporting Information). ^a)^τ_5_ value for Co in complex A, ^b)^τ_4_ value for Ni^2+^ complex B and C, ^c)^τ_4_ value for {Co(NO)_2_}^[^ ^10^ ^]^ in complexes B and C.

A stoichiometric amount of [Ni(NO)(CH_3_NO_2_)_3_][PF_6_]^[^ ^7^ ^]^ was added to a CH_3_CN solution of (dadt^Bz^)Co(NO) (dadt^Bz^ is *N,N*‐ dibenzyl‐3,7‐diazanonane‐1,9‐dithiolate) producing a color change within time of mixing at ca. 22 °C from dark purple to dark brown, concomitant with the ν(NO) IR changes that are displayed in **Figure** **1**. The absorption at 1603 cm^−1^ for ν(NO) of the precursor (dadt^Bz^)Co(NO) complex (Figure S2, Supporting Information) in CH_3_CN diminished as new bands at ca. 1652, 1780, and 1842 cm^−1^ appeared. Over the course of 4 h, the absorption bands at 1780 and 1842 cm^−1^ continued to increase while the absorption at 1652 cm^−1^ diminished (Figure 1). Upon addition of diethyl ether (Et_2_O), a brown crystalline product was isolated in ≈60% yield. While mass spectral results gave an elemental composition consistent with the targeted 1:1 adduct of [(dadt^Bz^)Co(NO)∙Ni(NO)]^+^ (Figure S5, Supporting Information), the X‐ray analysis of crystals showed a rearranged Co‐Ni product corresponding to the exchange of nickel for cobalt in the N_2_S_2_ binding site. A cobalt dinitrosyl complex, [(dadt^Bz^)Ni∙Co(NO)_2_][PF_6_] was crystallographically defined (**Figure** **2**, complex B).

The [(dadt^Bz^)Ni∙Co(NO)_2_]^+^ complex can also be synthesised following an alternate route by reacting the (dadt^Bz^)Ni (Figure S3, Supporting Information) with [(tmeda)Co(NO)_2_][BPh_4_]^[^ ^9^ ^]^ in DCM followed by crystallization from MeCN/Et_2_O (Figure 2, complex C). Several attempts to isolate and crystallize the intermediate for the reaction of [N_2_S_2_Co(NO)] with [Ni(NO)(CH_3_NO_2_)_3_]^+^ at −35 ˚C did not give good quality crystals, although a poor‐quality structure having trimetallic formulation of [{(dadt^Bz^)Co(NO)}_2_∙Ni][PF_6_]_2_ is given in Figure S11 (Supporting Information) as 20% probability thermal ellipsoid. We propose this trimetallic species to be a side product, rather than a reactive intermediate essential to the formation of the product from adduct, because such trimetallic species, as the [(N_2_S_2_)Co(NO)∙Ni∙(N_2_S_2_)Co(NO)]^2+^, are in thermodynamic valleys and found to be typically unreactive.

The molecular structures of (dadt^Bz^)Co(NO) (A), [(dadt^Bz^)Ni∙Co(NO)_2_][PF_6_] (B) and [(dadt^Bz^)Ni∙Co(NO)_2_][BPh_4_] (C) are shown in Figure 2 as 50% probability thermal ellipsoid plots; selected metric parameters are tabulated in Table S2 (Supporting Information). Complexes A and B crystallize in the same monoclinic *P2_1_/n* space group, while complex C crystallized in the monoclinic *C2/c* space group. The cobalt within the N_2_S_2_ binding site of (dadt^Bz^)Co(NO), adopts an almost ideal square pyramidal geometry with τ_5_ = 0.04. The displacement of the cobalt from the mean N_2_S_2_ plane is 0.298 Å. The apical NO molecule is severely bent, with ∠Co1‐N1‐O1 = 127.88˚. These parameters are consistent with those of several reported [(N_2_S_2_)Co(NO)] complexes with various hydrocarbon connectors between the heteroatoms.^[^ ^6^, ^10^ ^]^

The nickel center within the N_2_S_2_ pocket of [(dadt^Bz^)Ni∙Co(NO)_2_][PF_6_] (Complex **B**), is in a slightly distorted square planar geometry with τ_4_ = 0.14; the displacement of the nickel from the mean N_2_S_2_ plane is 0.056 Å. The Co of {Co(NO)_2_}^10^ is in a distorted tetrahedral geometry, τ_4_ = 0.87, and is coordinated by dissymmetric NO molecules (∠Co1‐N1‐O1 = 164.3˚ and ∠Co1‐N2‐O2 = 174.8˚). Two S atoms from the N_2_S_2_ ligand bridge Co and Ni. The latter produces a dihedral angle of intersection (hinge angle) between the S1‐Ni1‐S2 and S1‐Co1‐S2 planes of 108.04˚. In addition, the hinge angles establish a relatively short Ni‐Co distance of 2.803 Å. Based on the sum of the covalent atomic radii of Ni/Co, this M‐M’ distance is beyond bonding.

**Figure 3:**
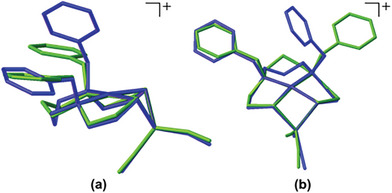
Overlay of the [(dadt^Bz^)Ni∙Co(NO)_2_]^+^ having two different anions PF_6_^−^ (Green, Complex **B**) and BPh_4_^−^ (Blue, Complex **C**). a) Side view b) Top view. OLEX2 was used for overlay. (Alignment RMSD: without inversion 1.596 Å and with inversion 1.571 Å.).

Crystallographic metric data for complex **C**, [(dadt^Bz^)Ni∙Co(NO)_2_][BPh_4_] are slightly different from complex **B**, [(dadt^Bz^)Ni∙Co(NO)_2_][PF_6_]. The major difference arises from the orientation of the benzylic phenyl rings. In the case of complex **B**, the rings are pointing away from each other, whereas in complex **C** the benzylic phenyl rings are pointing in the same direction, likely reflecting a packing difference between complex **B** and **C** (vide infra). The dihedral angle between the benzylic rings in complex **B** is found to be 7.61˚, whereas for complex **C** it is found to be 62.67˚ (Figure S12, Supporting Information). The crystal packing diagrams of complexes **B** and **C** are shown in Figures S13 and S14 (Supporting Information). Overlay of the [(dadt^Bz^)Ni∙Co(NO)_2_]^+^ structures from crystallization with two different anions PF_6_^−^ and BPh_4_^−^ is shown in **Figure** **3**. From DFT optimized structures of the two isomers using MeCN in the smd model, complex **B**, that with

the PF_6_^−^ anion (green in Figure 3), is found to be lower in energy compared to complex **C** (blue in Figure 3) having the BPh_4_^−^ anion by 2.5 kcal mol^−1^ (negligible). This supports the conclusion that packing differences in the two structures (complex **B** and **C**) give rise to differences in geometry and orientation of the benzylic phenyl rings.

The nitrosyl IR stretching frequencies as well as the M─N─O bond angle, and the M─N(O) and N─O bond lengths of reported cationic N donor and P donor cobalt dinitrosyl complexes are tabulated in Table S4 (Supporting Information). All are similar to the [(dadt^Bz^)Ni∙Co(NO)_2_]^+^ species. Despite the significant differences in geometrical orientation and Co‐N‐O angles in {Co(NO)_2_}^10^, the two NO units in Co(NO)_2_ are strongly coupled (2D IR, see Figure S15 Supporting Information) and best described as a unit, having symmetric and asymmetric stretching vibrations, similar to that of Fe(NO)_2_ in the [(N_2_S_2_)Ni∙Fe(NO)_2_]^+^ analogue.^[^ ^11^ ^]^

**Figure 4:**
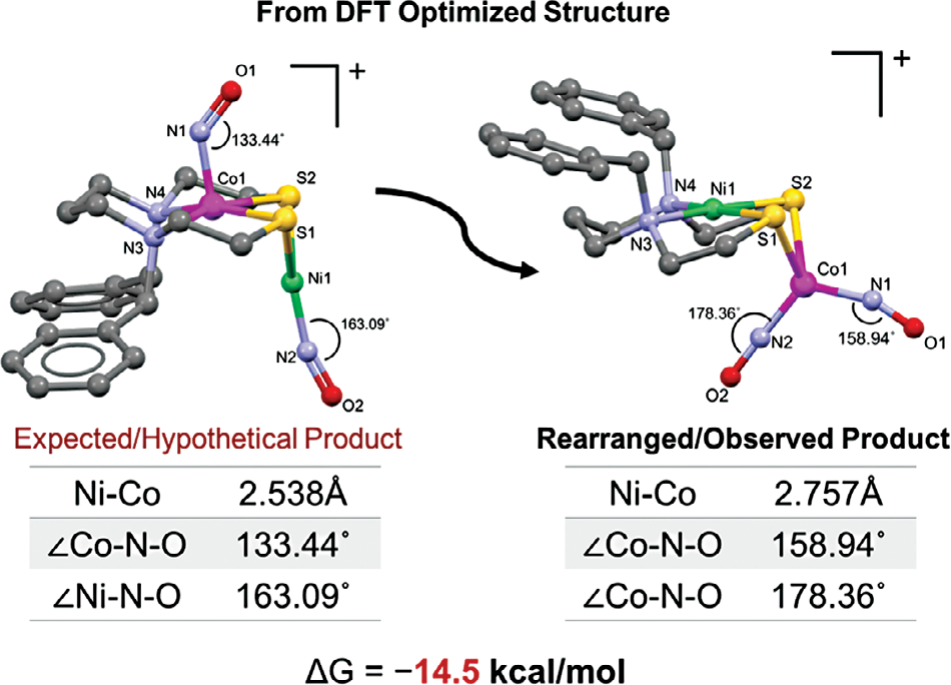
Energy difference between the expected/hypothetical and rearranged product as obtained from DFT calculations.

The fast, time of mixing, reaction of (dadt^Bz^)Co(NO) with [(CH_3_NO_2_)_3_Ni(NO)]^+^ suggest formation of an adduct, presumably the [(dadt^Bz^)Co(NO)∙Ni(NO)]^+^ analogous to Hayton's [(bipy)∙Ni(NO)]^+^ derivative,^[^ ^8^ ^a]^ expressed in **Figure** **4** as a DFT optimized structure. Via an unknown pathway of rearrangement, the adduct then forms the isolated product, in which Ni and Co have exchanged positions, along with NO transfer from Ni to Co, yielding the [(dadt^Bz^)Ni∙Co(NO)_2_]^+^ thermodynamic product. The redox changes would involve an overall two electron change, i.e., Ni^0^ to Ni^II^, and NO^+^ to NO^−^.

A computational study addressed the driving force for this rearrangement of [(dadt^Bz^)Co(NO)∙Ni(NO)]^+^ to [(dadt^Bz^)Ni∙Co(NO)_2_]^+^. The crystal structure of [(dadt^Bz^)Ni∙Co(NO)_2_]^+^ was imported to use as the starting coordinates for energy calculations, performed using TPSSTPSS^[^ ^12^ ^]^ functional and triple‐ζ basis set 6–311++G (d, p)^[^ ^13^ ^]^ in Gaussian 16 Revision C.01.^[^ ^14^ ^]^ All species were confirmed to be minimum energy structures by the absence of imaginary frequencies. The optimized structures of the expected/hypothetical product, and the isolated/rearranged product, in gas phase were then calculated using MeCN in the smd solvent model. The ground state energy difference, between these two structures (Figure 4) is found to be 14.5 kcal mol^−1^ in favour of the rearranged product.

**Scheme 1:**
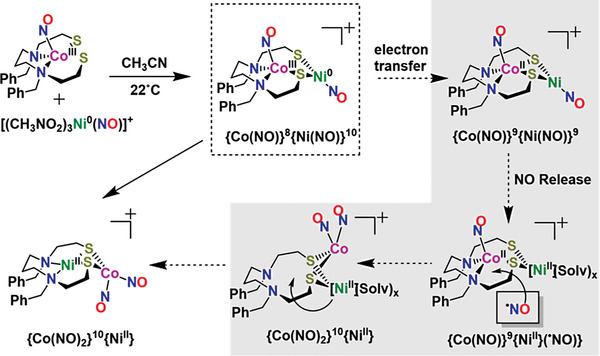
Probable steps for the formation of [(dadt^Bz^)Ni∙Co(NO)_2_][PF_6_] species. Including the presumed initial adduct, the species shown in grey background are hypothetical intermediates during the transformation.

To account for the reactivity observations, we pursue the hypothesis that the initial mixing of substrates generates a Co‐Ni species, presumably {Co(NO)}^8^{Ni(NO)}^10^ (**Scheme** **1**). A rapid electron transfer is expected to occur from {Ni(NO)}^10^ to {Co(NO)}^8^ to generate a {Co(NO)}^9^{Ni(NO)}^9^ species followed by NO release. The release of NO gas was detected by headspace analysis of the reaction mixture by gas chromatography at retention time of 2.56 min (Figure S25, Supporting Information). A similar observation was made by the Hayton group for the {Ni(NO)}^10^ unit in the [(bipy)Ni(NO)]^+^ or {Ni(NO)}^10^ complex which was found to release NO upon oxidation to {Ni(NO)}^9^.^[^ ^15^ ^]^ The control experiment found that separately the {Co(NO)}^8^ and {Ni(NO)}^10^ synthons did not show any NO release in MeCN, under similar reaction times (Figure S26, Supporting Information). The release of NO upon stirring and its attack on the {Co(NO)}^9^ would account for generation of {Co(NO)_2_}^10^ as it comes out of the N_2_S_2_ pocket. The resultant empty binding site is rapidly replaced by Ni(II) to form the stable square planar NiN_2_S_2_ complex.

The role of released NO during the reaction is supported by the significant increase in the isolated yield of the final product (from 15% to 60%), when the reaction is carried out in a septum‐sealed vial in order to inhibit NO loss. However, there is an optimal NO concentration as performing the reaction under excess pressure of exogeneous or added NO gas considerably decreased the rate of the reaction as well as the amount of product formation (see Figure S22, Supporting Information). Based on these observations, NO release during the reaction is likely involved in the rate determining step for the rearrangement that forms the final product. The mechanism expressed in Scheme 1 is consistent with these results, including the increased yield in the closed system. However, coupling between the NO molecules has not been observed or indicated during the course of the reaction.

**Scheme 2:**
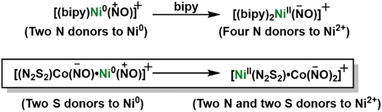
Analogy between bidentate bipyridine (reported by Hayton's group^[^ ^7^, ^8^, ^15^ ^]^) and nitrosylated metallodithiolate as ligands for the {Ni(NO)}^10^ synthon.

The oxidation state ambiguity of the {Ni(NO)}^10^ ‐unit (Enemark‐Feltham notation) as expressed within the Hayton's bipyridine complexes,^[^ ^7^, ^8^, ^15^ ^]^ indicates a two‐electron redistribution of oxidation state when a second bipyridine is added to the tri‐coordinated, [(bipy)Ni(NO)]^+^, as shown below in **Scheme** **2**. In our case, the four donors within the N_2_S_2_ tetradentate ligand are well known to stabilize Ni^II^. The thermodynamic downhill rearrangement reaction promotes, in a bimetallic fashion, the two‐electron oxidation state change for nickel. The transfer of ∙NO and an electron (overall NO^−^) to {Co(NO)}^8^, generating the {Co(NO)_2_}^10^, maintains the overall electron balance, but requires an impressive amount of oxidation state redistribution. That is, the chemical non‐innocence of the N_2_S_2_Co(NO) metallodithiolate ligand supports the rearrangement of Ni^0^/Ni^2+^ by an intramolecular bimetallic process. Further computational studies using the CASSCF valance bond analysis method is required to estimate the amount of NO^+/∙/−^ character in each complex of Scheme 2.^[^ ^16^ ^]^

## 3 Conclusion

In conclusion, the (dadt^Bz^)Co(NO) metalloligand reacts with a [Ni(NO)]^+^ synthon to form an unstable intermediate within the time of mixing but undergoes rearrangement slowly (ca. 4 h) at room temperature to yield a thermodynamically stable heterobimetallic Ni(N_2_S_2_) cobalt dinitrosyl species. Such ligand rearrangement/reconstitution is not possible with the immobile/rigid bipyridine ligand framework and Ni^2+^ must be stabilized by an extra bipyridine ligand.^[^ ^8^ ^a]^ While there are several cobalt dinitrosyl complexes reported in the literature,^[^ ^9^, ^17^ ^]^ to the best of our knowledge this is the first reported heterobimetallic cobalt dinitrosyl complex (DNCC). The identity of the original adduct can only be surmised; however, a Co/Ni interchange is most likely resulting from intramolecular process evolving from the first formed adduct. The rearranged product, [Ni∙Co(NO)_2_]^+^ requires a formal overall transfer of an NO^−^ species, or an NO‐coupled electron transfer (NOCET). The polarizability of the electron density between metal and NO ligands strengthens the suggested role of metal carriers in NO capture and its transfer, i.e., regulation which is ultimately critical in biological NO chemistry.

## Conflict of Interest

The authors declare no conflict of interest.

## Supporting information

Supporting Information

## Data Availability

The data that support the findings of this study are available in the supplementary material of this article.
