# Supplementary material for: Bond Trading: Intramolecular Metal and Ligand Exchange within a NO/Ni/Co Complex
Source: Adv Sci (Weinh). 2023 Dec 3;11(6):2307113. doi: 10.1002/advs.202307113 (PMC10853699; doi:10.1002/advs.202307113)
Supplement: Supplementary file 1 — Supporting Information [file ADVS-11-2307113-s001.pdf]

ADVANCED SCIENCE
Open Access

Supporting Information

for *Adv. Sci.*, DOI 10.1002/advs.202307113

Bond Trading: Intramolecular Metal and Ligand Exchange within a NO/Ni/Co Complex

*Manish Jana, Xueyan Zheng, Trung Le, Manuel Quiroz, Paulina Guererro-Almaraz, Donald J. Darensbourg and Marcetta Y. Darensbourg**

Bond Trading: Intramolecular Metal and Ligand Exchange within a NO/Ni/Co Complex

by

Manish Jana[a], Xueyan Zheng[a], Trung Le[a], Manuel Quiroz[a], Paulina Guererro-Almaraz[a], Donald J. Darensbourg[a] and Marcetta Y. Darensbourg*[a]

[a] *Department of Chemistry*

Texas A&M University

College Station, TX 77843

Email: marcetta@chem.tamu.edu

Supporting Information

S1: ***Experimental Section:***

S1.1 ***Methods and Materials.*** All reactions and manipulations were performed either under inert atmosphere N_2 glove box or using standard Schlenk-line and syringe/rubber septa techniques under N_2 atmosphere. Dry solvents were purified and degassed via a Bruker solvent system. Reagents were purchased from commercial sources and used as received. The known compound $[Ni(NO)(CH_3NO_2)_3][PF_6]$[1] was synthesized following the literature reported procedure.

S1.2 Physical measurements. Solution and solid infrared spectra were recorded on a Bruker Tensor 37 Fourier transform IR (FTIR) spectrometer in CaF_2 solution cell with a 0.2 mm path length and as thin films, respectively. ^1H and ^{13}C NMR spectra were recorded using the Bruker Avance NEO 400 MHz system with an automated tuning 5mm broadband iProbe (Acend magnet). Electrospray ionization mass spectrometry (ESI-MS) was performed by the Laboratory for Biological Mass Spectrometry at Texas A&M University.

Single crystal X-ray diffraction measurements were carried out at a low temperature employing a (three circle or kappa) Bruker-AXS (Quest or Venture) with IμS source and a Photon III area detector diffractometer for (Mo Kα radiation, λ = 0.71073 Å or Cu Kα radiation, λ = 1.54178 Å) (NSF-CHE-9807975, NSF-CHE-0079822 and NSF-CHE-0215838). Crystals (for color, habit, size see corresponding CIF files) were mounted on a nylon or Kapton® loops and cooled in a cold nitrogen stream (OXFORD Cryosystems (700 or 800), to 110(2) K. Bruker AXS APEX 3[2] software was used for data collection and reduction. Absorption corrections were applied using SADABS.[3] Space group assignments were determined by examination of systematic absences, E-statistics, and successive refinement of the structures. Structures were solved using SHELXT[4] and refined by least-squares refinement on F^2 followed by difference Fourier synthesis (OLEX2, SHELXL).[5,6] All hydrogen atoms were included in the final structure factor calculation at idealized positions and were allowed to ride on the neighboring atoms with relative isotropic displacement coefficients. Thermal parameters were refined anisotropically for all non-hydrogen atoms to convergence. The MERCURY 2022.1.0[7] interface was used for structure visualization, analysis of bond distances and angles, and drawing ORTEP plots. OLEX2[6] interface was used for structure overlay.

S1.3 Cyclic Voltammetry. Cyclic voltammograms were recorded on a CHI600E electrochemical analyzer (HCH instruments, Inc.). All experiments were performed under an Ar blanket in CH_3CN solutions containing a 0.1 M $[^nBu_4N][PF_6]$ electrolyte and 2.0 mM analyte at room temperature. A 0.071 cm^2 glassy carbon disk was used as the working electrode, platinum wire as the counter electrode, and Ag^0/AgCl as the reference electrode. All potentials were referenced to the Fc/Fc^+ couple at 0.00 V.

S1.4 ***Synthesis of the ligand (N,N'-dibenzyl-3,7 diazanonane-1,9-dithiolate), abbreviated as dadt^{Bz} (1).***
A 100 mL long-necked Schlenk flask containing a magnetic stir bar is connected to a Schlenk line containing an overpressure valve, and the flask is flushed with N_2. The flask is charged with N, N' dibenzylpropylenediamine (2.54 g, 10 mmol) and dry toluene (40 mL). Ethylene sulfide (1.5 mL, 25 mmol) is added by syringe, and the solution is stirred for 48 h at 80°C.
If a white precipitate develops, the cooled solution should be anaerobically filtered through Celite in a glass-fritted funnel. The solvent was removed in vacuum while maintaining the temperature between 60 and 65°C. This resulted a pale yellow to colourless liquid (3.25 g, 87%). ^1H NMR (CD_3CN): δ 1.5 (quint, H, CH_2, J = 4Hz), δ 1.66 (s, 2H, SH), δ 2.31 (t, 2H, CH_2, J = 4Hz), δ 2.38 (t, 2H, CH_2, J = 5Hz), δ 2.44 (t, 2H, CH_2, J = 7Hz), δ 2.48 (t, 2H, CH_2, J = 7Hz), δ 3.58 (s, H, CH_2), δ 7.15 (t, 6H, CH, J = 8Hz), δ 7.2 (d, 4H, CH, J = 4Hz).

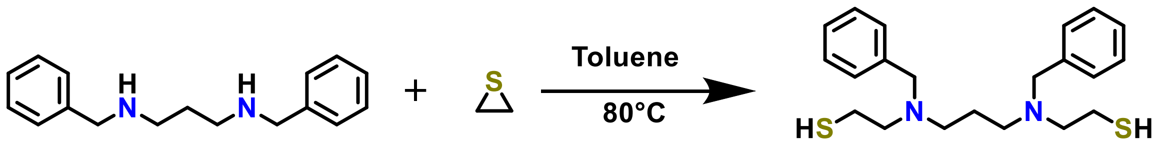

S1.5 ***Synthesis of [Co(dadt)]₂.*** 1.1 g (0.0028 mmol) of dadt[Bz] ligand is taken up in 5 mL of toluene and a suspension of 0.686 g (0.0026 mmol) anhydrous cobalt(II) acetylacetonate, $Co(AcAc)_2$ in toluene is added to it. (The ligand is taken in slight excess as it is readily soluble in ether or toluene and can be removed by washing with ether or toluene.) The reaction is allowed to stir under inert atmosphere overnight and the color changes from dark purple to dark blue green with formation of solids. The crude solid is collected on a Büchner funnel under anaerobic conditions. The solid is washed thoroughly with toluene and ether to remove the unreacted ligand. Yield: 1.05 g (44%). This was used for nitrosylation without further purification and characterization.

S1.6 ***Synthesis of Co(dadt^{Bz})(NO).*** In a 20 mL scintillation vial, 1 g (1.1 mmol) of $[Co(dadt^{Bz})]_2$ was dissolved in minimum amount of DCM and NO gas was passed through it. The color of the solution changed from dark blue to dark brown. Ether was added to the reaction mixture after stirring for 1h at room temperature. Addition of ether yields dark brown solid. The solid is collected on a Büchner funnel under anaerobic conditions and washed with 10mL ether twice. The solids were dissolved in minimum volume of dichloromethane and pentane was layered very carefully and kept inside −35˚C freezer to yield crystals suitable for x-ray diffraction. Yield: 0.591 g (58 %).

Anal. Calcd. for $C_{21}H_{28}N_3CoS_2O$ (F.W. = 461.528): C, 54.65; H, 6.12; N, 9.1. Found: C, 54.18; H, 5.98; N, 8.71.

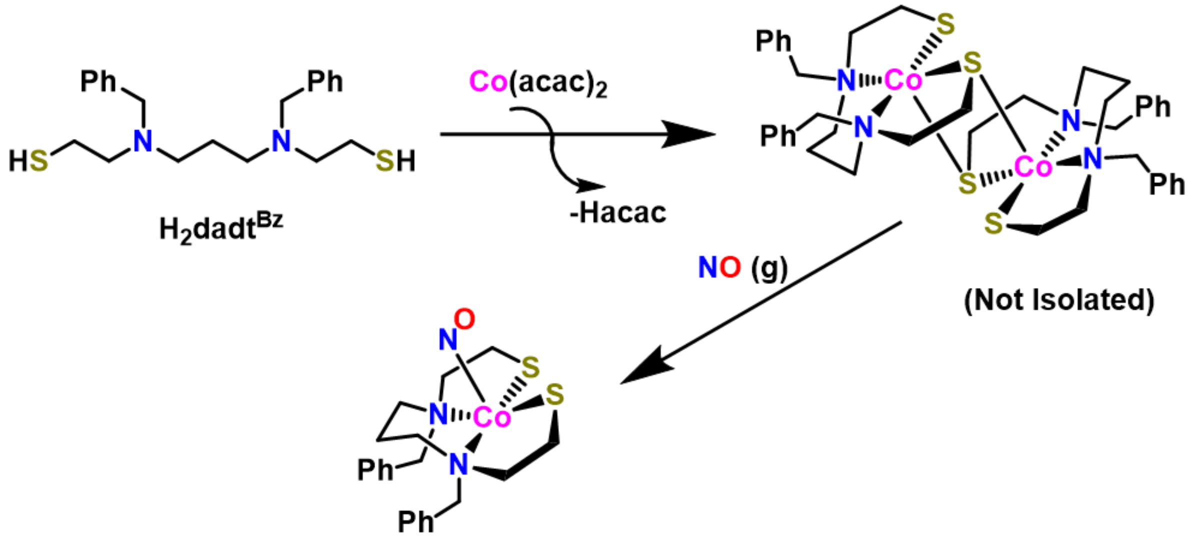

Properties: UV–vis spectrum in DCM [λ_{max} (nm) (ε ($M^{-1}cm^{-1}$))]: 498 (768±41), 386 (3126±37), 307 (12892±680), 265 (13418±1549). Mass Spectrum *m/z* (experimental) $[M+H]^+$ = 462.1069 (Calculated m/z = 462.1079).

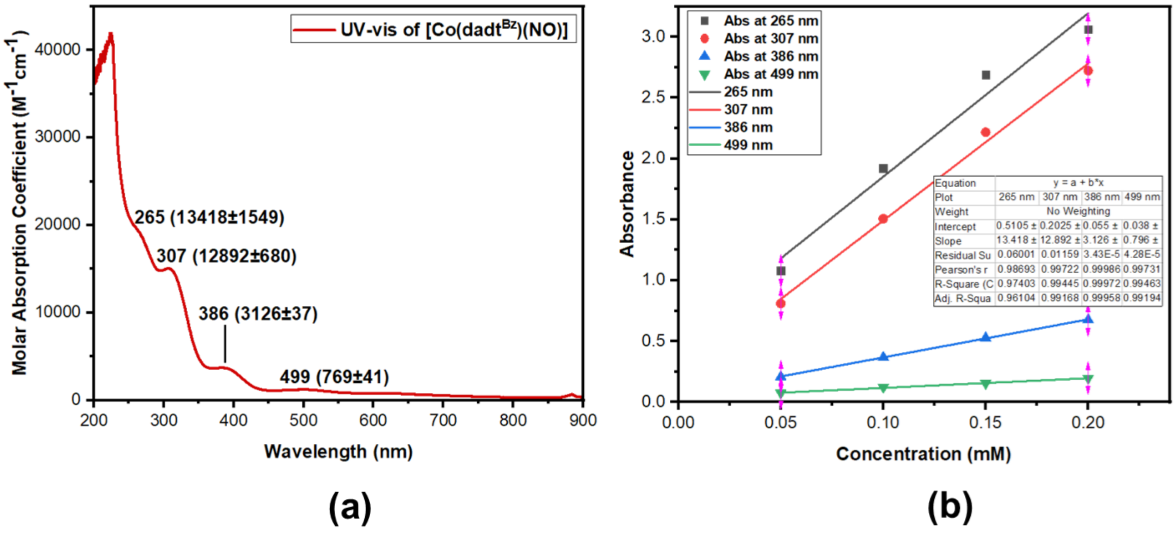

Figure S1: UV Vis spectra of (dadt^{Bz})Co(NO) in MeCN. (B) Beer–Lambert plot of (dadt^{Bz})Co(NO) in MeCN.

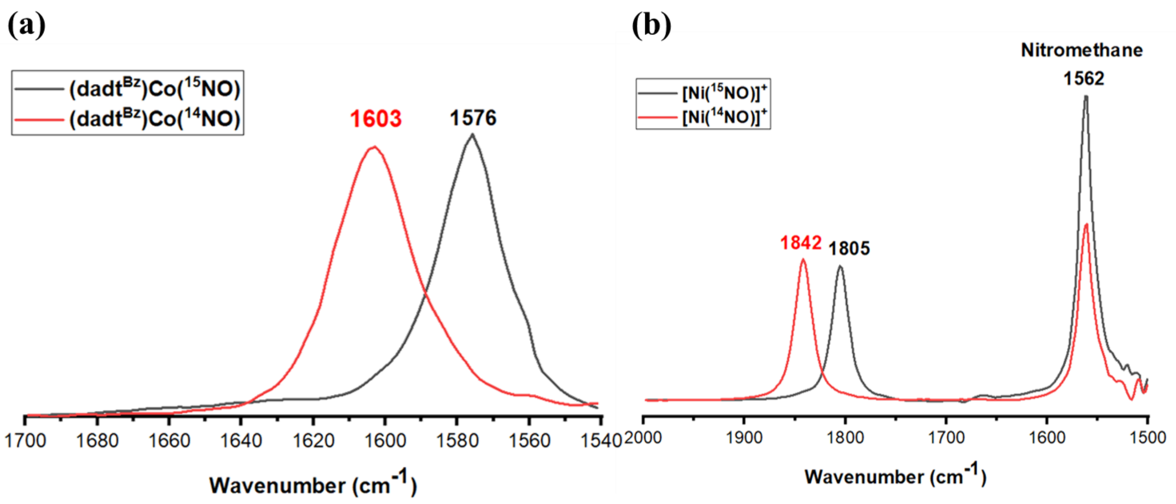

Figure S2: Solution IR Spectrum of (a) (dadt^{Bz})Co(^{14}NO) (red) and its ^{15}NO analogue (black) in MeCN. (b) [Ni(^{14}NO)(CH_3NO_2)]$^+$ (red) and its ^{15}NO analogue (black) in MeCN.

S1.7 *Synthesis of Ni(dadt^{Bz}).* 1 g (0.0026 mmol) of dadt^{Bz} ligand was taken in 5 mL of toluene and a suspension of 0.668 g (0.0026 mmol) Ni(OAcAc)$_2$ in toluene was added to it. The reaction was allowed to stir under inert atmosphere overnight and the colour changed from green to dark brown with formation of solids. The crude solid was collected on a Büchner funnel under anaerobic conditions. The solid was filtered and washed thoroughly with toluene and ether. Yield: 0.998 g (89 %). Crystals suitable for x-ray diffraction were obtained from layering concentrated dichloromethane solution with hexane.

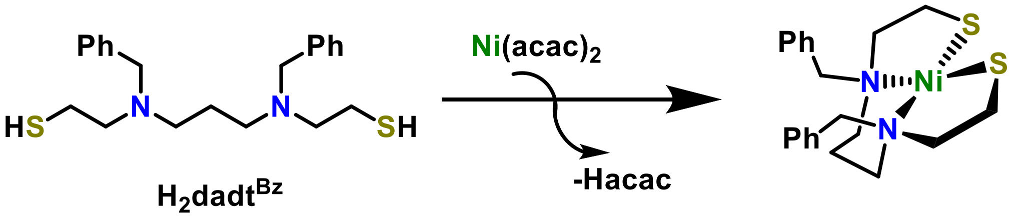

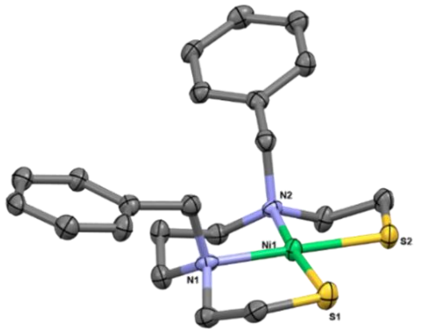

Ni-S	2.17Å
Ni-N	2.024Å
S-Ni-S	84.11˚
N-Ni-N	97.67˚
Ni_{disp}-(N_2S_2)	0.007 Å
τ_4	0.10

Figure S3: Single crystal X-ray structure for the Ni(dadt^{Bz}) species in 50% probability thermal ellipsoid plot. Hydrogen atoms were omitted for clarity.

Table S1 Crystal data and structure refinement for Ni(dadt^{Bz}).

CCDC number	**2261462**
Identification code	MJ_Ni_dadt_0m
Empirical formula	$C_{21}H_{28}N_2NiS_2$
Formula weight	431.28
Temperature/K	110.0
Crystal system	Monoclinic
Space group	$P2_1/c$
a/Å	8.9483(4)
b/Å	12.3455(5)
c/Å	18.3265(8)
α/°	90
β/°	102.834(2)
γ/°	90
Volume/Å^3	1973.97(15)
Z	4
ρ_{calc}g/cm^3	1.451
μ/mm^{-1}	3.435
F(000)	912.0
Crystal size/mm^3	0.5 × 0.5 × 0.3
Radiation	CuKα (λ = 1.54178)
2Θ range for data collection/°	8.706 to 133.526
Index ranges	-10 ≤ h ≤ 10, -14 ≤ k ≤ 14, -21 ≤ l ≤ 21
Reflections collected	53670
Independent reflections	3497 [R_{int} = 0.0925, R_{sigma} = 0.0292]
Data/restraints/parameters	3497/0/235
Goodness-of-fit on F^2	1.083
Final R indexes [I>=2σ (I)]	R_1 = 0.0689, wR_2 = 0.1254
Final R indexes [all data]	R_1 = 0.0954, wR_2 = 0.1463
Largest diff. peak/hole / e Å^{-3}	0.73/-0.56

S1.8 ***Synthesis of [Ni(dadt^{Bz})•Co(NO)$_2$]PF$_6$.*** Under inert atmosphere glove box, 83.3 mg (0.2 mmol) of [Ni(NO)(CH$_3$NO$_2$)$_3$]PF$_6$ was taken in a 20mL scintillation vial and a suspension of (dadt^{Bz})Co(NO) 91.2 mg (0.2 mmol) in 5 mL of MeCN was added to it and the vial was capped with septum. The reaction was allowed to stir overnight at room temperature. After stirring overnight, the reaction mixture was filtered through celite and layered with ether at room temperature resulting in block shaped crystals after one day. Yield: 60%. (Spectroscopic yield = 78% as determined from the IR, see Figure S4 for details.) Mass spectrum is given in Figure S5. Solid IR (ν_{NO}) = 1820 and 1760 cm^{-1} (Figure S6), Solution IR in MeCN (ν_{NO}) = 1842 and 1780 cm^{-1}.

Anal. Calcd. for C$_{21}$H$_{28}$N$_4$CoS$_2$O$_2$NiPF$_6$ (F.W. = 695.192): C, 36.28; H, 4.06; N, 8.06. Found: C, 36.22; H, 4.21; N, 8.37.

^{1}H and ^{13}C NMR is given in Figures S8 and S9 respectively.

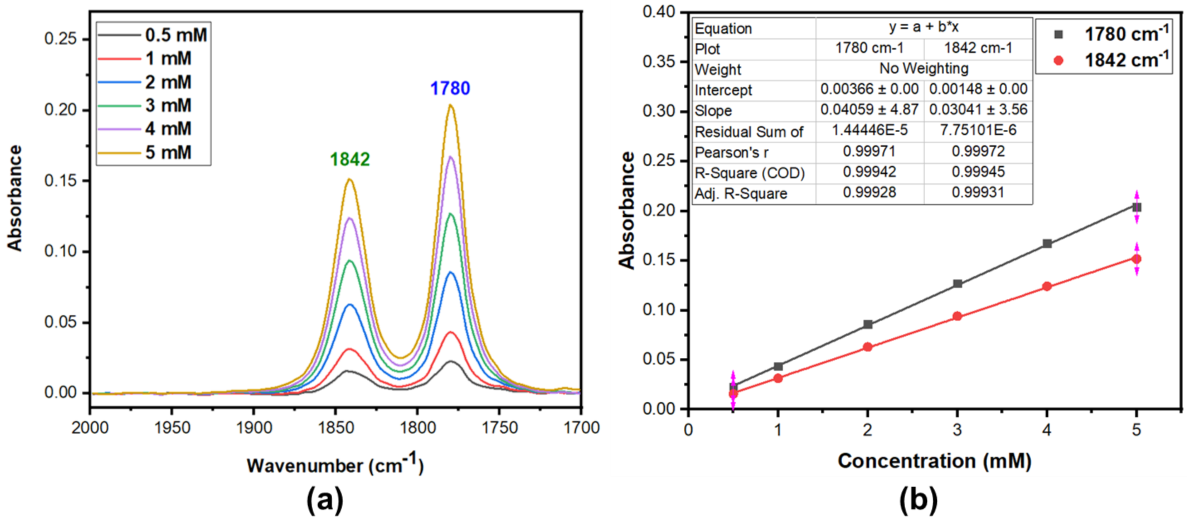

Figure S4: (a) Solution IR spectrum of the [(dadt^{Bz})Ni•Co(NO)$_2$]PF$_6$ at different concentrations. (b) Plot of absorbance vs concentration at 1780 and 1842 cm^{-1}.

From Figure S4,

For absorbance at 1780 cm^{-1},

$$absorbance = 0.04059 * (concentration) + 0.00366$$

For absorbance at 1842 cm^{-1},

$$absorbance = 0.03041 * (concentration) + 0.00148$$

The spectroscopic yield calculated from the reaction between (dadt^{Bz})Co(NO) and [Ni(NO)]$^+$ is found to be 78%.

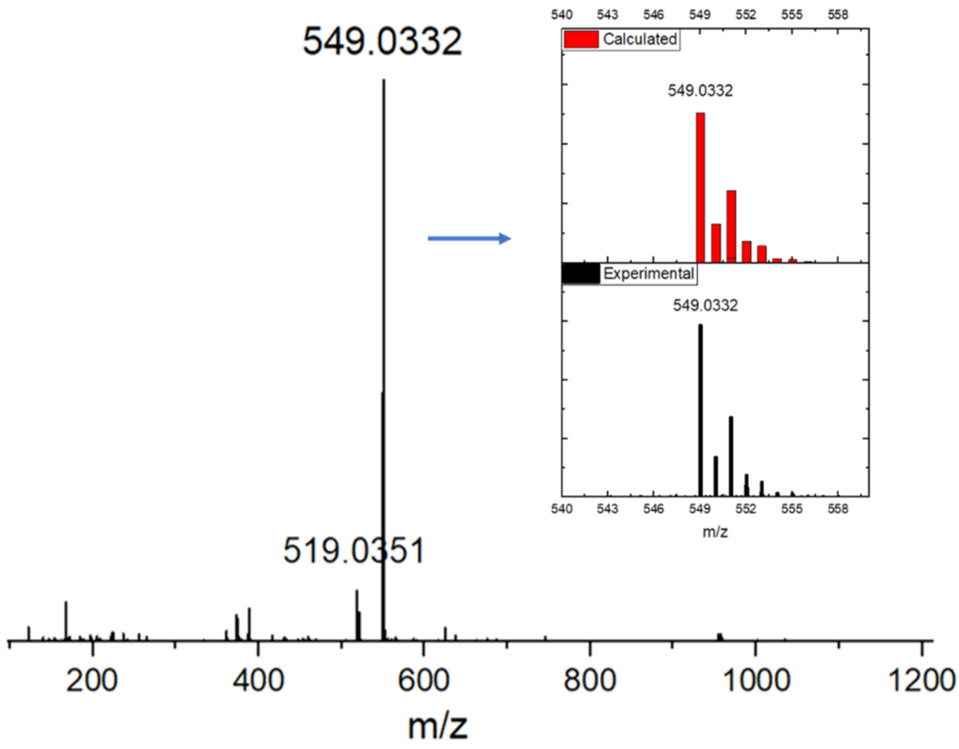

Figure S5: Mass spectrum for product from the reaction between (dadt^{Bz})Co(NO) and $[Ni(NO)(CH_3NO_2)_3]^+$ showing the presence of the $[(dadt^{Bz})Ni{\bullet}Co(NO)_2]^+$ as parent peak (m/z = 549.0332 (experimental), m/z = 549.0332 (calculated)). The peak at m/z = 519.0351 (experimental) (m/z = 519.0359 (calculated)) correspond to $[M-(NO)]^+$.

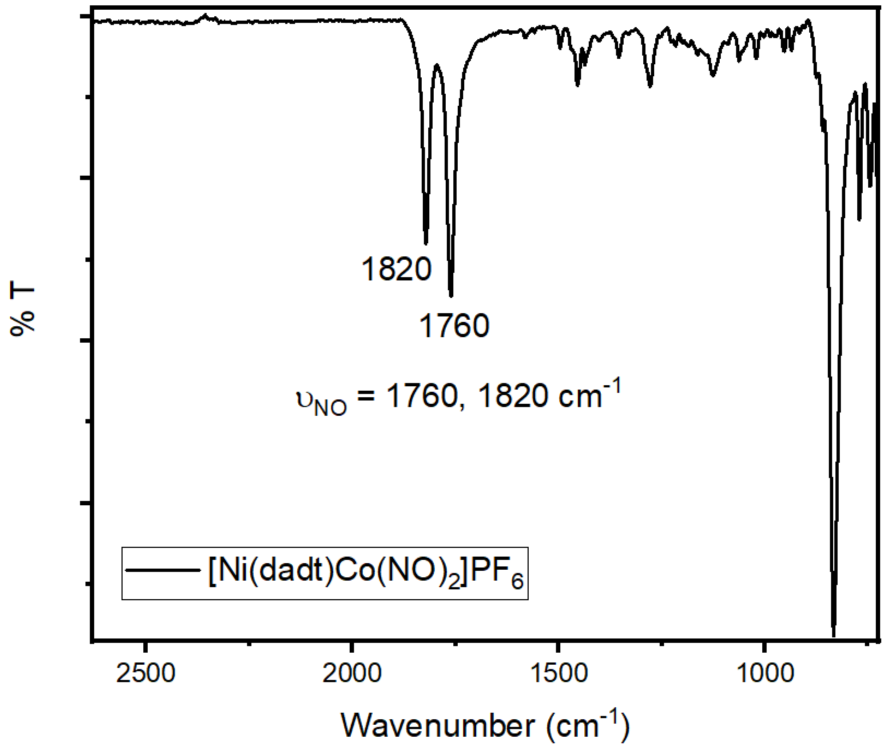

Figure S6: Solid state IR of $[(dadt^{Bz})Ni{\bullet}Co(NO)_2](PF_6)$.

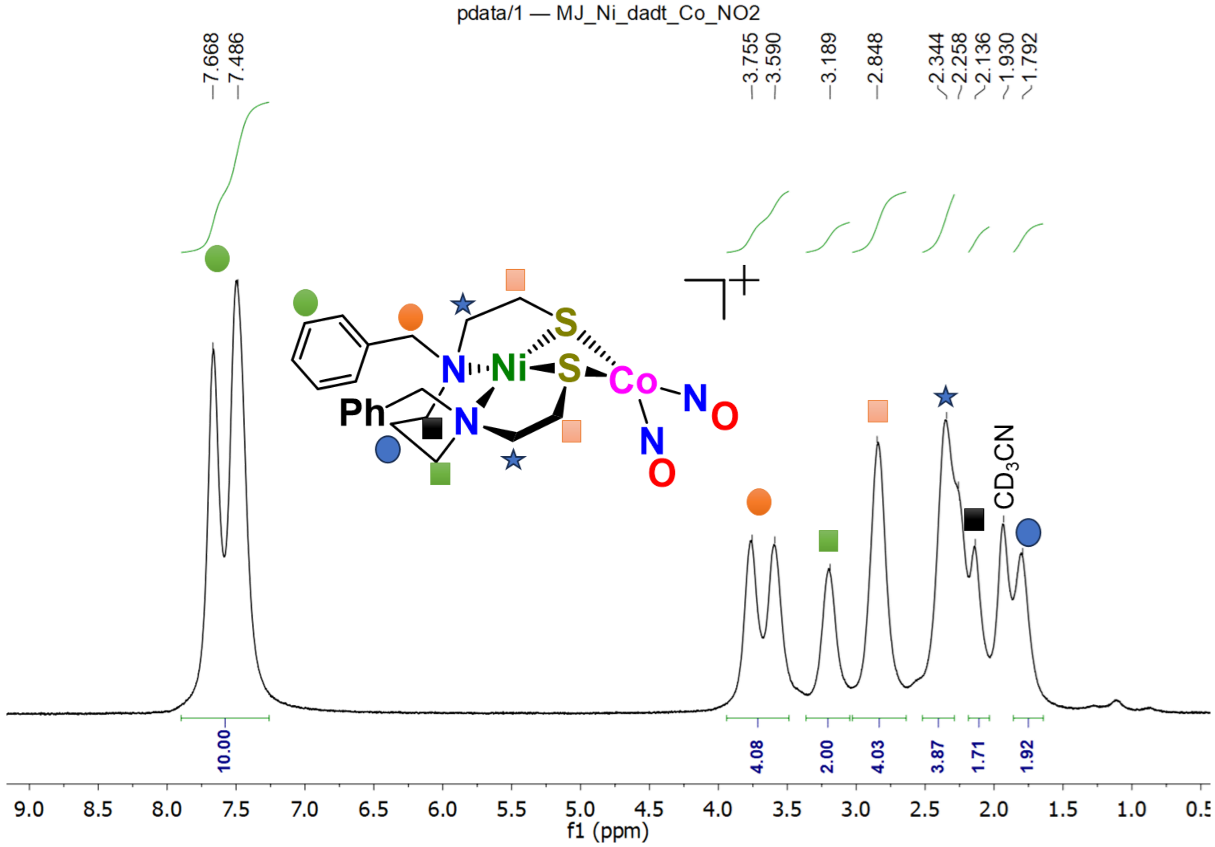

Figure S7: ^{1}H NMR Spectrum of $[(dadt^{Bz})Ni{\bullet}Co(NO)_2]^+$ in CD_3CN.

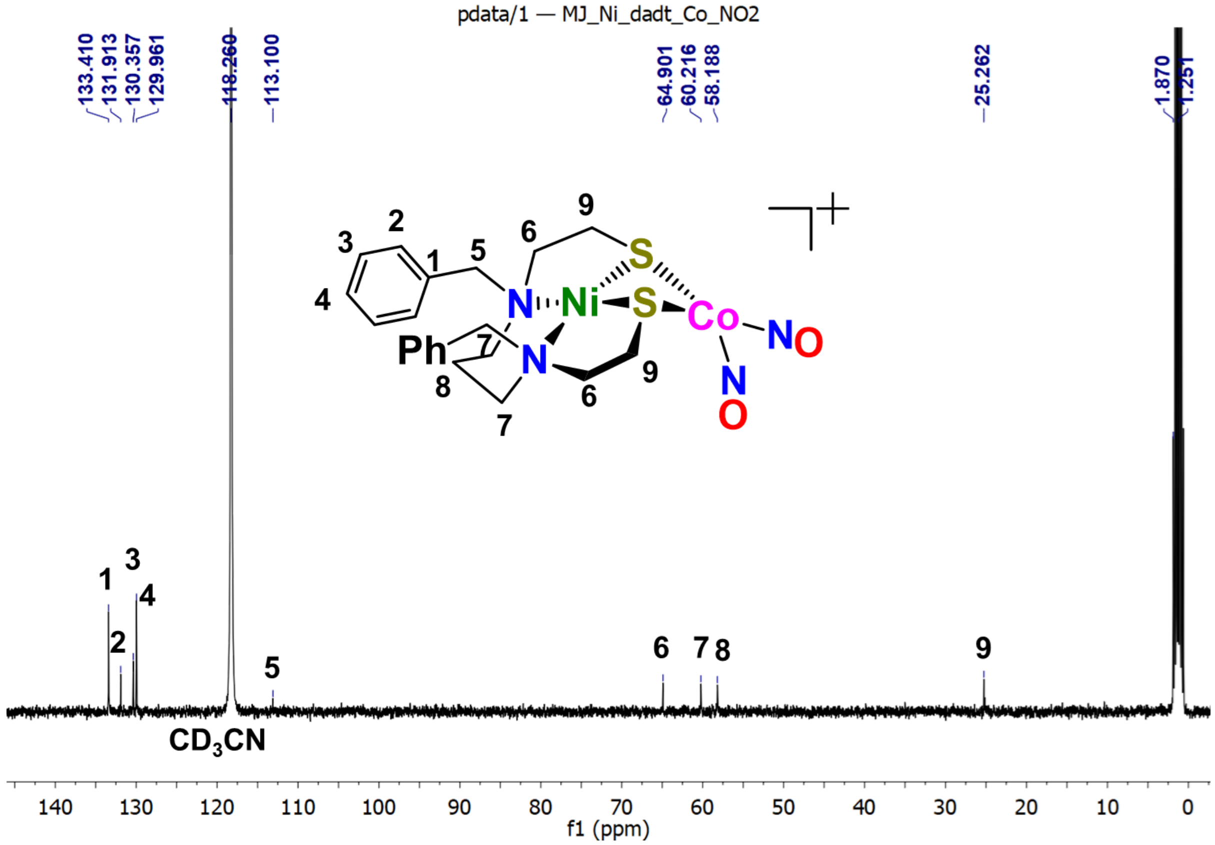

Figure S8: ^{13}C NMR Spectrum of $[(dadt^{Bz})Ni{\bullet}Co(NO)_2]^+$ in CD_3CN.

S2. Scrambling Experiment:

Scrambling experiment was performed using a mixture of Co(^{15}NO) and [Ni(^{14}NO)]$^+$ in MeCN at room temperature. In the final product, (given in Figure S9b and S10) mixture of all the possible dinitrosyl species, [Ni•Co(NO)$_2$]$^+$ were found (^{14}NO and ^{15}NO, determined from mass spectrometry and IR spectroscopy).

In a separate experiment, when the final products i.e; [Ni•Co(^{14}NO)$_2$]$^+$ with [Ni•Co(^{15}NO)$_2$]$^+$ were mixed together in MeCN, they were found to be scrambling in solution as well (see Figure S9b). Hence, which complex [Co(NO) or Ni(NO)] is releasing NO during the reaction could not be determined from the scrambling experiment.

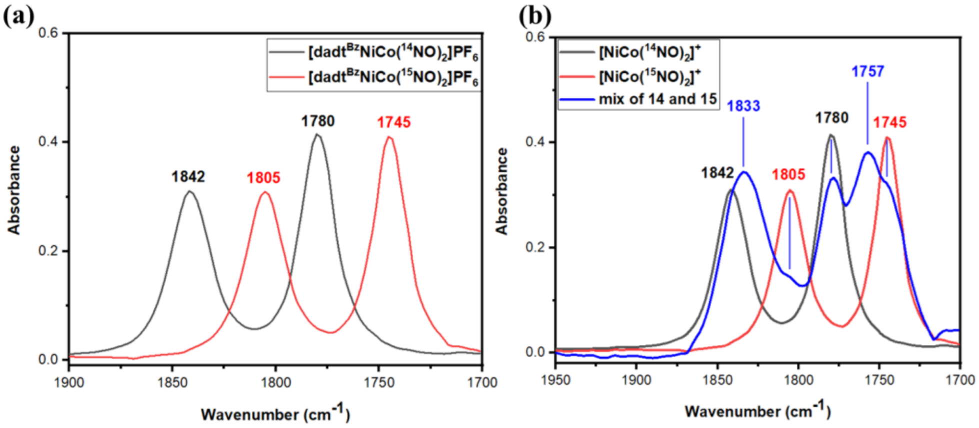

Figure S9: (a) Solution IR spectrum of [(dadt^{Bz})Ni•Co(^{14}NO)$_2$]$^+$ (black) and [(dadt^{Bz})Ni•Co(^{15}NO)$_2$]$^+$ (red) in MeCN. (b) Solution IR spectrum (blue) of a mixture of [(dadt^{Bz})Ni•Co(^{14}NO)$_2$]$^+$ and [(dadt^{Bz})Ni•Co(^{15}NO)$_2$]$^+$ in MeCN, showing scrambling between ^{14}NO and ^{15}NO in solution to give mixture of products.

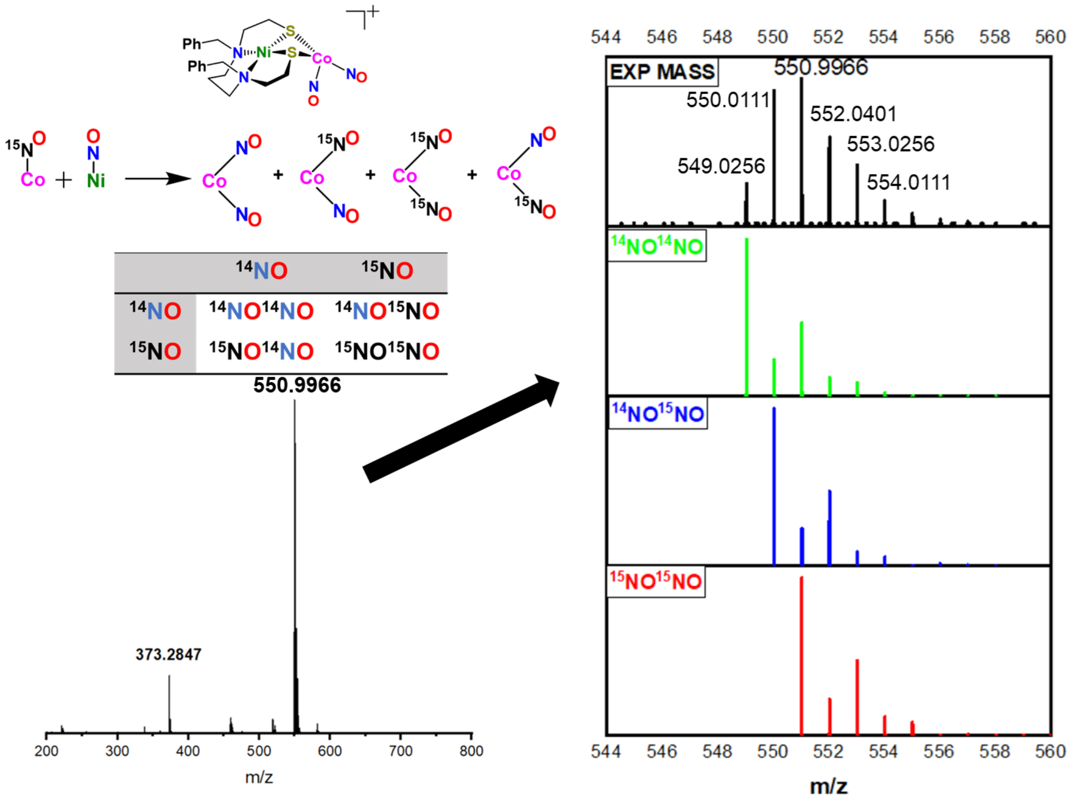

Figure S10: Mass spec for the reaction of (dadt^{Bz})Co(^{15}NO) with [(CH$_3$NO$_2$)$_3$Ni(^{14}NO)]$^+$ showing the scrambling of NO in the final product.

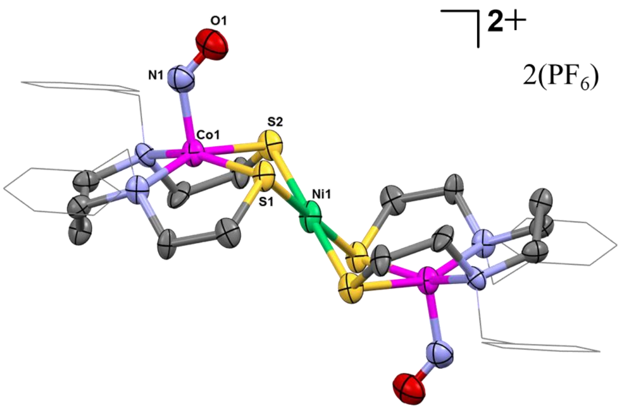

∠Co1-N1-O1	130.8 ˚
∠S1-Co1-S2	79.89 ˚
∠S1-Ni1-S2	81.78 ˚
Co1-Ni1	3.073 Å
Co1-N1	1.755 Å
N1-O1	1.10 Å

Figure S11: Single crystal structure of [{(dadt^{Bz})Co(NO)}$_2$•Ni](PF_6)$_2$ isolated at −35˚C in 20% probability thermal ellipsoid. Hydrogen atoms are omitted for clarity. Despite several attempts we were unable to grow good quality crystals.

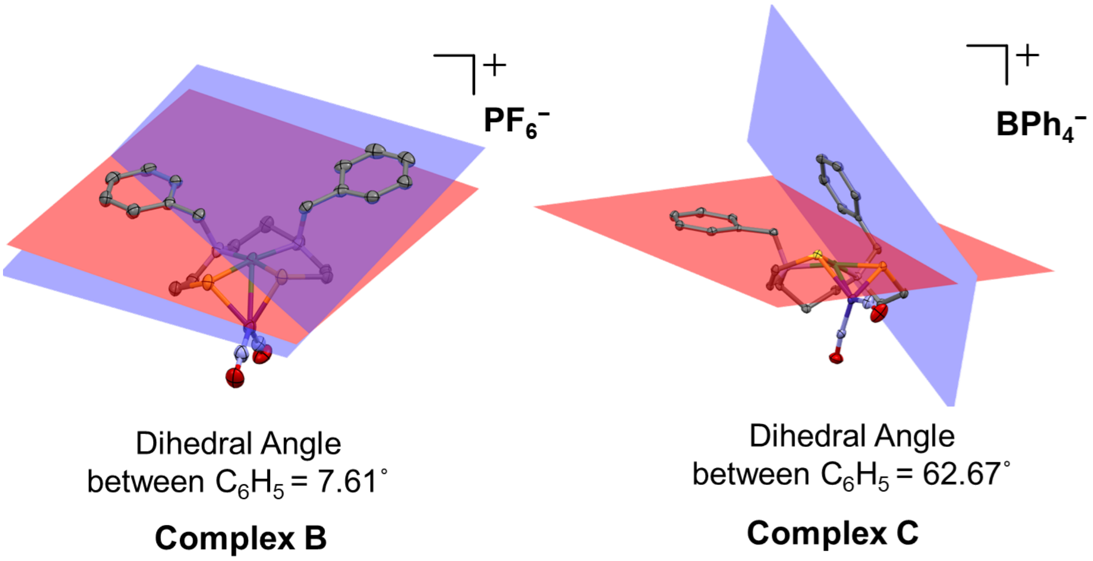

Figure S12: Dihedral angle between the two benzylic rings in Complex **B** and **C**.

Crystal Packing Diagram:

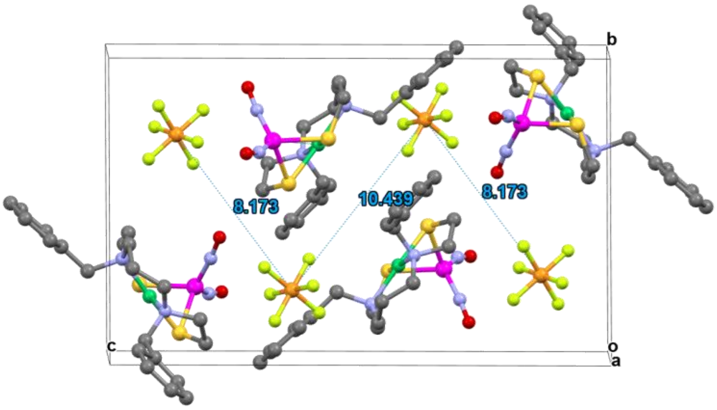

Figure S13: Crystal packing diagram of [(dadt^{Bz})Ni•Co(NO)$_2$][PF_6]. Hydrogen atoms are omitted for clarity.

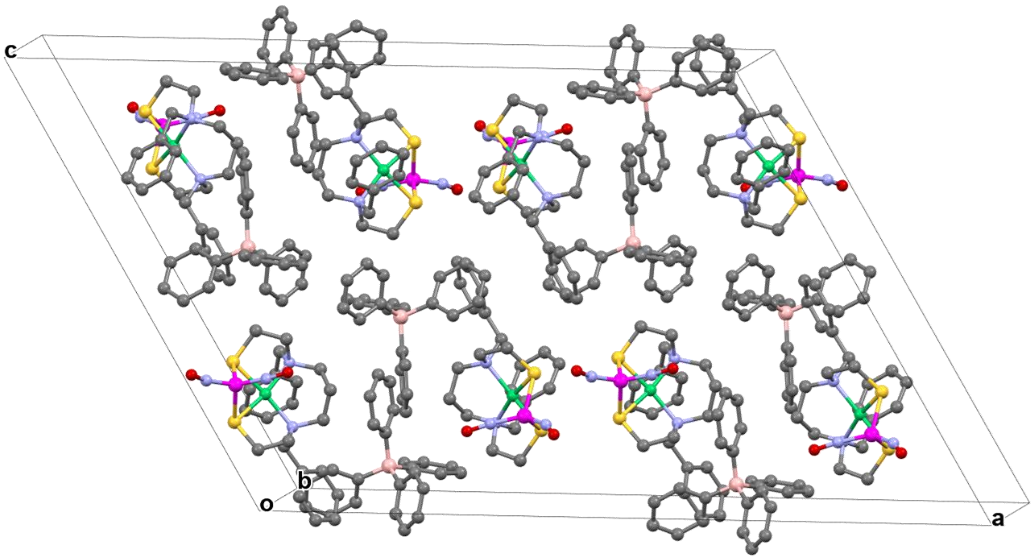

Figure S14: Crystal packing diagram of [(dadt^{Bz})Ni•Co(NO)$_2$][BPh$_4$]. Hydrogen atoms are omitted for clarity.

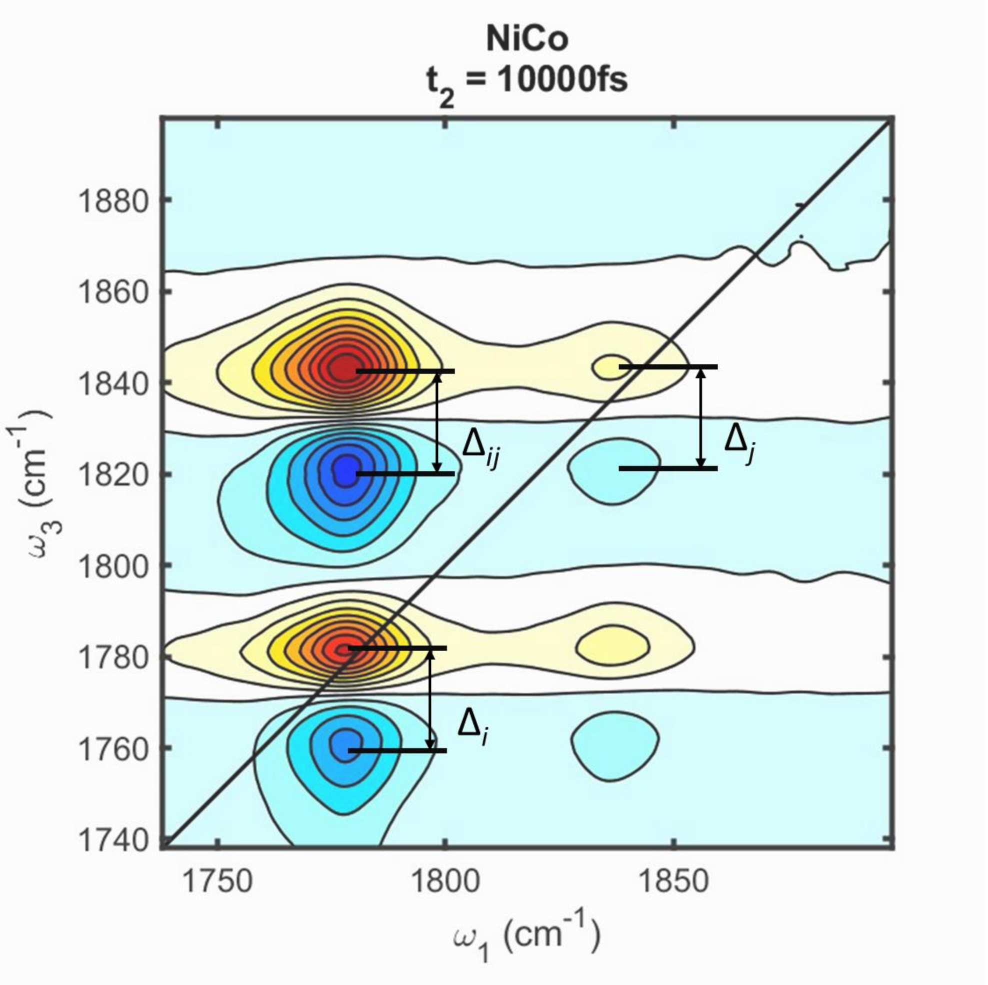

Figure S15: 2D IR of [(dadt^{Bz})Ni•Co(NO)$_2$][PF$_6$] confirms that NO molecules are strongly coupled. Where, $\Delta_i = \Delta_j$ = 21 cm^{-1} (anharmonicity) and Δ_{ij} = 21 cm^{-1} (cross-anharmonicity).

S3. *Kinetic Studies:*

The strong NO stretching band provides a convenient spectroscopic handle to monitor the formation of metal nitrosyl species by IR spectroscopy. The cobalt nitrosyl complex has an intense IR absorption maximum at 1603 cm^{-1}, well separated from the absorption maximum of the reactant nickel nitrosyl, 1842 cm^{-1} (Figure S2). Upon mixing the $[Ni(NO)]^+$ synthon with the $(dadt^{Bz})Co(NO)$ in MeCN, a reaction occurs within the time of mixing and bands corresponding to the starting material disappeared (Figure S16a, green line).

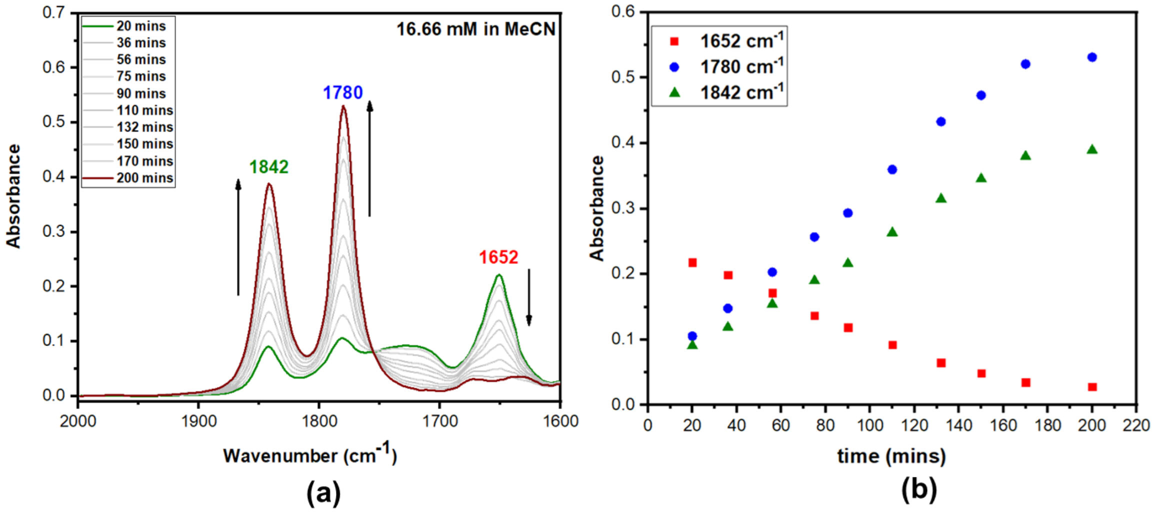

Figure S16: (a) The NO stretching region of IR spectra collected at room temperature during the reaction of $[Co(NO)(dadt^{Bz})]$ with $[Ni(NO)(CH_3NO_2)_3]^+$ in MeCN. The initial concentration of each compound was 16.66 mM. The band at 1652 cm^{-1} corresponds to ν_{NO} of presumed intermediate. This signal decayed within 200 min as a new band at 1780 cm^{-1} and 1842 cm^{-1} emerged, indicating formation of $[Ni(dadt^{Bz})Co(NO)_2]^+$. (b) The change in absorbance with time for the absorption bands at 1652 cm^{-1} (red), 1780 cm^{-1} (blue) and 1842 cm^{-1} (green).

However, new absorptions at 1652 and 1780 cm^{-1} grew in as well as a new band that overlaps with the 1842 cm^{-1} of the Ni(NO) starting material was observed (Figure S2b). The decrease in the absorbance of the intermediate peak at 1652 cm^{-1} and the growth of absorption features at 1780 cm^{-1} and 1842 cm^{-1} over the course of the reaction were monitored by ν(NO) IR spectroscopy (Figure S16a) at different temperatures. An isosbestic point at 1756 cm^{-1} indicated the interconversion of only two species. The change in the absorbance behaviour i.e., the decrease and increase in the absorption of NO bands (ν_{NO}) with time are shown in Figure S16b**.** A plot of (absorbance)/(absorbance)$_{max}$] with respect to time gave a straight line with R^2 = 99%, whereas the natural log plot of the same gave a straight line with R^2 = 97% as shown in Figure S17.

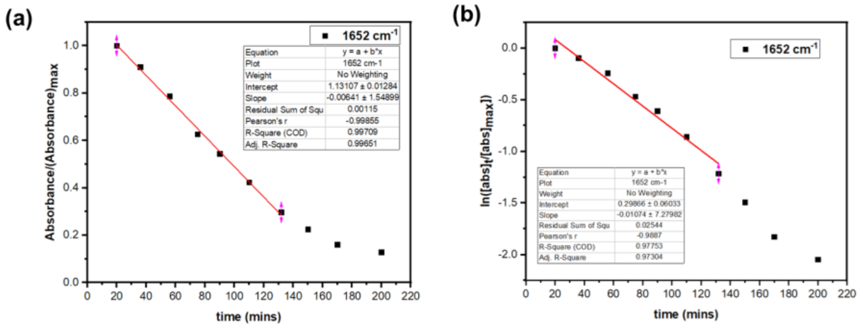

Figure S17: (a) Plot of (Absorbance)/(Absorbance)$_{max}$ with time (R^2 = 99%) and (b) natural log plot of (absorbance)/(absorbance)$_{max}$ with time (R^2 = 97%) monitoring the disappearance of the band at 1652 cm^{-1}.

The overall reaction can be written as:

$$Co(NO)(dadt^{Bz}) + [Ni(NO)(CH_3NO_2)_3]^{+} \xrightarrow{t.o.m} \begin{matrix} \text{Intermediate} \\ + \\ NO \end{matrix} \longrightarrow \text{Product}$$

Rate = k*[Intermediate]*[NO]

The reaction appears to be zero order with respect to intermediate concentration because either it is present in great excess during the reaction or intermediate is the site in which the reaction occurs. Once all the intermediate molecules are occupied, additional nitric oxide molecules must wait until the decomposition of the intermediate molecule occurs to form product. The presence of the free NO gas during the reaction has been identified by GC-MS from the reaction headspace.

Kinetic studies at different temperatures:

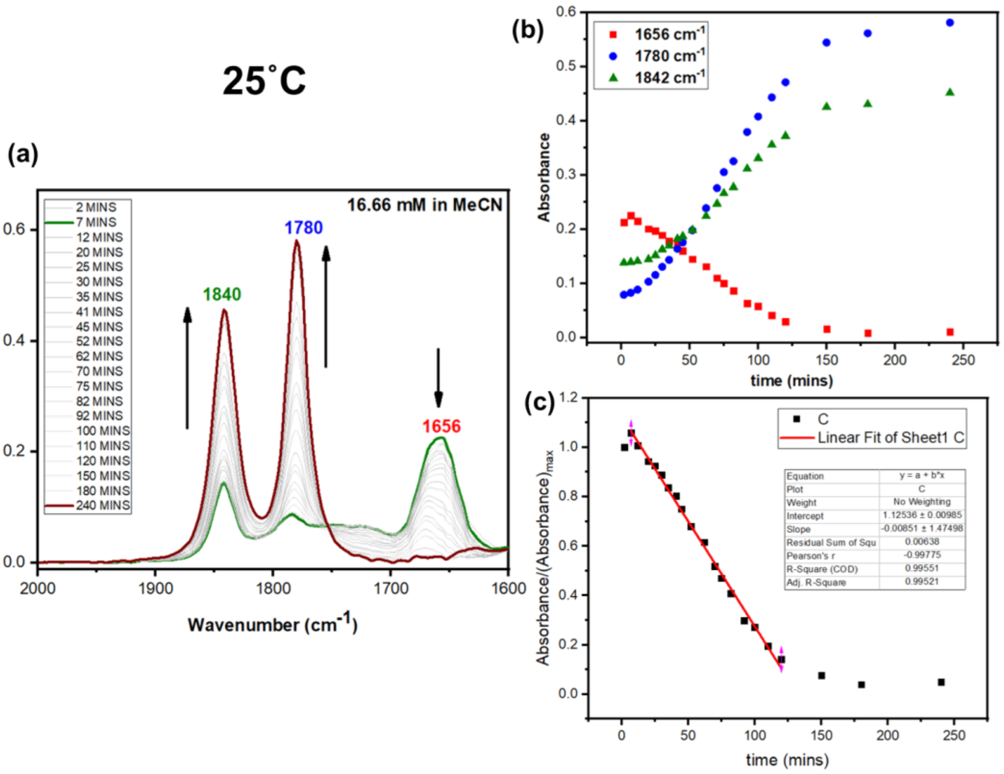

Figure S18: (A) Infrared monitoring of nitrosyl stretching frequency during the reaction of $(dadt^{Bz})Co(NO)$ and $[Ni(NO)(CH_3NO_2)_3]^{+}$ at 25˚C. (B) Reaction profiles of the ν_{NO} infrared bands for the conversion of intermediate species to the product. (**Red**: Intermediate species, **Blue and Green**: Product) (C) Mole fraction ((absorbance/(absorbance)$_{max}$) vs time of the ν_{NO} bands, monitoring the disappearance of the intermediate band at 1656 cm^{-1}. From the plot slope = 0.00851 mM min^{-1}.

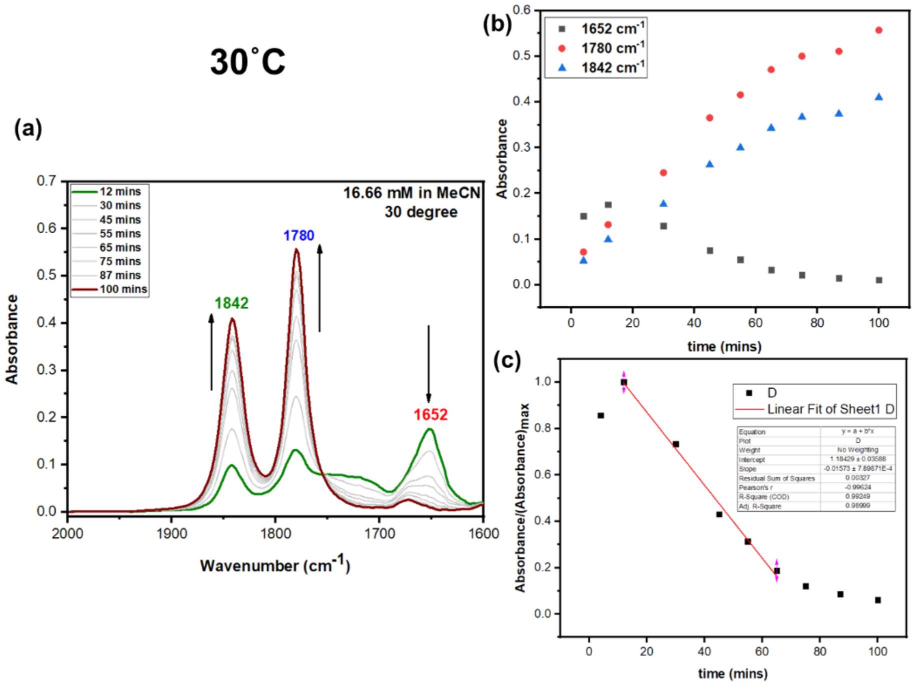

Figure S19: (A) Infrared monitoring of nitrosyl stretching frequency during the reaction of (dadt^{Bz})Co(NO) and $[Ni(NO)(CH_3NO_2)_3]^+$ at 30˚C. (B) Reaction profiles of the ν_{NO} infrared bands for the conversion of intermediate species to the product. (**Red**: Intermediate species, **Blue and Green**: Product) (C) Mole fraction ((absorbance)/(absorbance)$_{max}$) vs time of the ν_{NO} bands, monitoring the disappearance of the intermediate band at 1652 cm^{-1}. From the plot slope = 0.01573 mM min^{-1}.

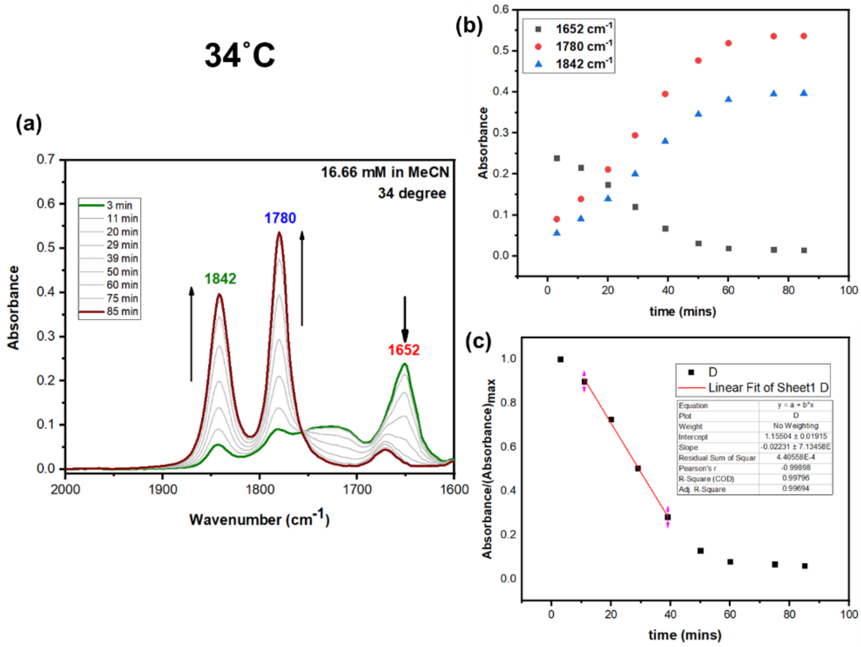

Figure S20: (A) Infrared monitoring of nitrosyl stretching frequency during the reaction of (dadt^{Bz})Co(NO) and $[Ni(NO)(CH_3NO_2)_3]^+$ at 34˚C. (B) Reaction profiles of the ν_{NO} infrared bands for the conversion of intermediate species to the product. (**Red**: Intermediate species, **Blue and Green**: Product) (C) Mole fraction ((absorbance)/(absorbance)$_{max}$) vs time of the ν_{NO} bands, monitoring the disappearance of the intermediate band at 1652 cm^{-1}. From the plot slope = 0.02231 mM min^{-1}.

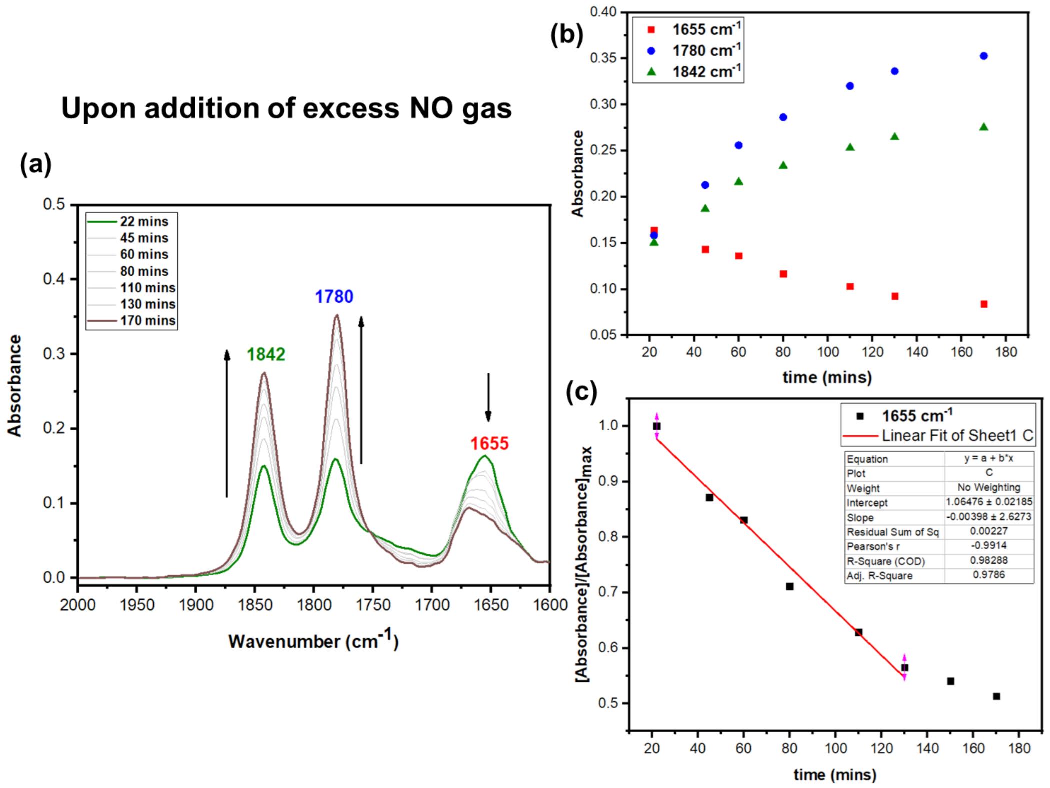

Figure S21: (A) Infrared monitoring of nitrosyl stretching frequency during the reaction of $(dadt^{Bz})Co(NO)$ and $[Ni(NO)(CH_3NO_2)_3]^+$ in presence of excess NO gas at RT. (B) Reaction profiles of the ν_{NO} infrared bands for the conversion of intermediate species to the product. (**Red**: Intermediate species, **Blue and Green**: Product) (C) Mole fraction ((absorbance)/(absorbance)$_{max}$) vs time of the ν_{NO} bands, monitoring the disappearance of the intermediate band at 1655 cm^{-1}. From the plot slope = 0.0038 mM min^{-1}.

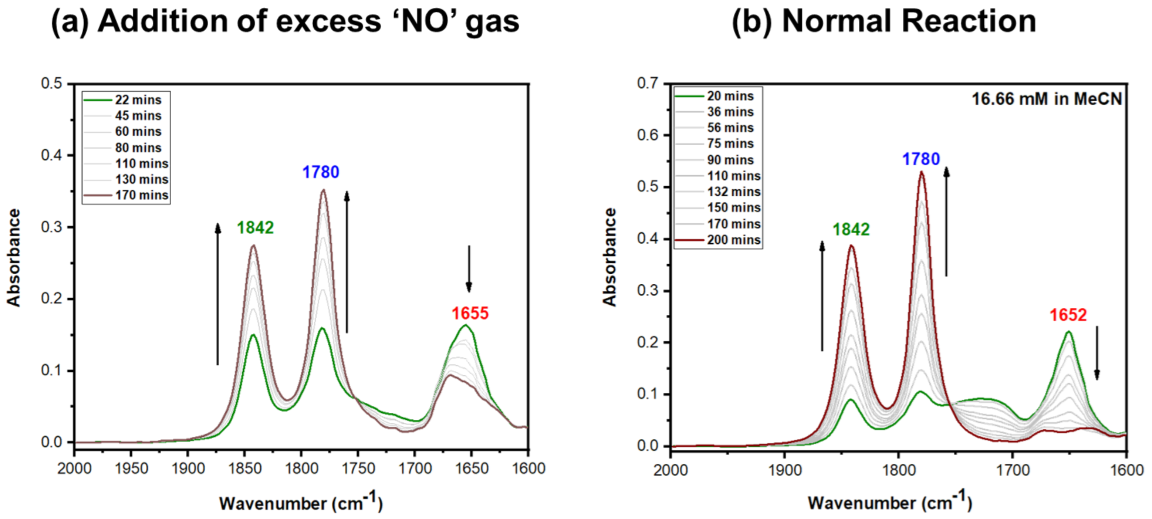

Yield of the product = 51%
(after 3 hrs)

Yield of the product = 76 %
(after 3 hrs)

Figure S22: The progress of the reaction (a) in presence of excess NO gas (b) in absence of any added NO gas. The yield of the product in each case has been determined from the calibration curve given in Figure S4.

Table S2: Selected bond lengths and angles of (dadt^{Bz})Co(NO) (the free metallodithiolate ligand) and [(dadt^{Bz})Ni•Co(NO)$_2$]$^+$.

	**[Co(NO)] (A)**	**[NiCo(NO)$_2$][PF$_6$] (B)**	**[NiCo(NO)$_2$][BPh$_4$] (C)**
		**MN$_2$S$_2$^{a}**	
M-N-O/(˚)	127.9(2)	-	-
M-NO/(Å)	1.773(3)	-	-
S-M-S/(˚)	85.36(3)	81.47(3)	82.71(3)
N-M-N/(˚)	96.36(8)	101.05(9)	102.03(7)
M-N$_2$S$_{2disp}$/(Å)	0.298	0.056	0.109
τ	0.04^c	0.14^d	0.16^d
		**Co(NO)$_2$^{b}**	
Co-N1-O1/(˚)	-	164.3(3)	160.9(2)
Co-N2-O2/(˚)	-	174.8(2)	174.82(19)
Co-N1/(Å)	-	1.682(3)	1.676(2)
Co-N2/(Å)	-	1.652(3)	1.654(2)
S1-M-S2/(˚)	-	76.18(3)	78.29(3)
N1-M-N2/(˚)	-	121.95(13)	117.63(10)
τ	-	0.87^d	0.90^d
Hinge^e /(˚)	-	108.04	108.71
M-M/(Å)	-	2.803	2.842

^{a} Metric data for MN$_2$S$_2$ unit, ^{b} Metric Data for Co(NO)$_2$ unit, ^{c} τ_5 value, ^{d} τ_4 value. ^{e} The angle of intersection between the S1-Ni1-S2 and S1-Co1-S2 planes.

Table S3: Crystal data and structure refinement for (dadt^{Bz})Co(NO) (**A**), [(dadt^{Bz})Ni•Co(NO)$_2$]PF_6 (**B**) and [(dadt^{Bz})Ni•Co(NO)$_2$]BPh_4 (**C**).

CCDC number	**2208117**	**2195743**	**2264676**
Identification code	(dadt^{Bz})Co(NO)	[(dadt^{Bz})Ni•Co(NO)$_2$]PF_6	[(dadt^{Bz})Ni•Co(NO)$_2$]BPh_4
Empirical formula	$C_{21}H_{28}CoN_3OS_2$	$C_{21}H_{28}CoF_6N_4NiO_2PS_2$	$C_{45}H_{48}BCoN_4NiO_2S_2$
Formula weight	461.51	695.20	869.44
Temperature/K	110.0	110.0	110.00
Crystal system	monoclinic	Monoclinic	monoclinic
Space group	$P2_1/n$	$P2_1/n$	C2/c
a/Å	12.6644(9)	9.9885(5)	35.151(6)
b/Å	13.2374(10)	12.7350(6)	10.7055(17)
c/Å	12.7823(9)	20.9145(10)	24.607(5)
α/°	90	90	90
β/°	101.199(2)	96.597(2)	120.009(9)
γ/°	90	90	90
Volume/Å^3	2102.1(3)	2642.8(2)	8018(2)
Z	4	4	8
ρ_{calc}g/cm^3	1.458	1.747	1.440
μ/mm^{-1}	1.032	8.502	1.032
F(000)	968.0	1416.0	3632.0
Crystal size/mm^3	0.3 × 0.3 × 0.2	0.1 × 0.1 × 0.05	0.5 × 0.5 × 0.3
Radiation	MoKα (λ = 0.71073)	CuKα (λ = 1.54178)	MoKα (λ = 0.71073)
2Θ range for data collection/°	4.142 to 50.176	8.142 to 133.408	4.742 to 50.684
Index ranges	-15 ≤ h ≤ 15, -15 ≤ k ≤ 15, -15 ≤ l ≤ 15	-11 ≤ h ≤ 11, -15 ≤ k ≤ 15, -24 ≤ l ≤ 24	-42 ≤ h ≤ 42, -12 ≤ k ≤ 12, -29 ≤ l ≤ 29
Reflections collected	86136	70671	84137
Independent reflections	3726 [R_{int} = 0.0933, R_{sigma} = 0.0286]	4675 [R_{int} = 0.0746, R_{sigma} = 0.0281]	7299 [R_{int} = 0.0598, R_{sigma} = 0.0264]
Data/restraints/parameters	3726/0/253	4675/0/343	7299/0/505
Goodness-of-fit on F^2	1.073	1.090	1.132
Final R indexes [I>=2σ (I)]	R_1 = 0.0339, wR_2 = 0.0675	R_1 = 0.0351, wR_2 = 0.0917	R_1 = 0.0351, wR_2 = 0.0643
Final R indexes [all data]	R_1 = 0.0502, wR_2 = 0.0761	R_1 = 0.0402, wR_2 = 0.0943	R_1 = 0.0405, wR_2 = 0.0660
Largest diff. peak/hole / e Å^{-3}	0.63/-0.44	0.46/-0.47	0.36/-0.30

Table S4: Dinitrosyl cobalt complexes reported in literature.

	ν(N–O)/cm^{-1}	M–N(O)/Å	N–O/Å	M–N–O/ °	Reference
[(dadt^{Bz})Ni•Co(NO)$_2$]PF$_6$	1840 (MeCN)	1.652(3)	1.153(3)	174.8(2)	This Work
	1780 (MeCN)	1.682(3)	1.164(3)	164.3(3)	
[Co(NO)$_2$(L1")](BF$_4$)	1875 (CH$_2$Cl$_2$)	1.654(5)	1.151(7)	175.8(7)	Ref 8
	1798 (CH$_2$Cl$_2$)	1.673(6)	1.137(10)	164.6(7)	
[Co(NO)$_2$(py)$_2$](BF$_4$)	1876 (CH$_2$Cl$_2$)	1.654(6)	1.130(8)	170.2(6)	Ref 9
	1798 (CH$_2$Cl$_2$)	1.644(6)	1.156(8)	170.1(6)	
[Co(NO)$_2$(tmeda)](BPh$_4$)	1866 (KBr)	1.6630(12)	1.1475(16)	168.53(13)	Ref 10
	1789 (KBr)	1.6636(12)	1.1582(15)	165.83(11)	
[Co(NO)$_2$(dppe)](PF$_6$)	1860 (Nujol)	1.656 (10)	1.130 (14)	176.6(14)	Ref 11
	1790 (Nujol)	1.671 (11)	1.142 (13)	172.3(11)	
[Co(NO)$_2$(PPh$_3$)$_2$]BPh$_4$	1851 (KBr)	1.645 (6)	1.174 (6)	171.0 (5)	Ref 12
	1790 (KBr)	1.645 (6)	1.174 (6)	171.0 (5)	

L1" = bis(3,5-diisopropyl-1-pyrazolyl)methane, SPh = benzenethiolate, Ar-nacnac = anion of [(2,6-diisopropylphenyl)NC(Me)]$_2$CH, tmeda = *N,N,N',N'*-tetramethylethylenediamine.

S4. *Electrochemical Studies:*

Cyclic voltammograms, Figure S23, of (dadt^{Bz})Co(NO) (**A**) and [(dadt^{Bz})Ni•Co(NO)$_2$]PF$_6$(**B**) were recorded in CH$_3$CN solution using glassy carbon as a working electrode, Ag wire as a reference electrode and Pt wire as a counter electrode, containing 0.1M [^{n}Bu$_4$N][PF$_6$] as the supporting electrolyte at room temperature under argon and referenced to Fc$^{+/0}$ ($E_{1/2}$ = 0.0V) as an internal standard.

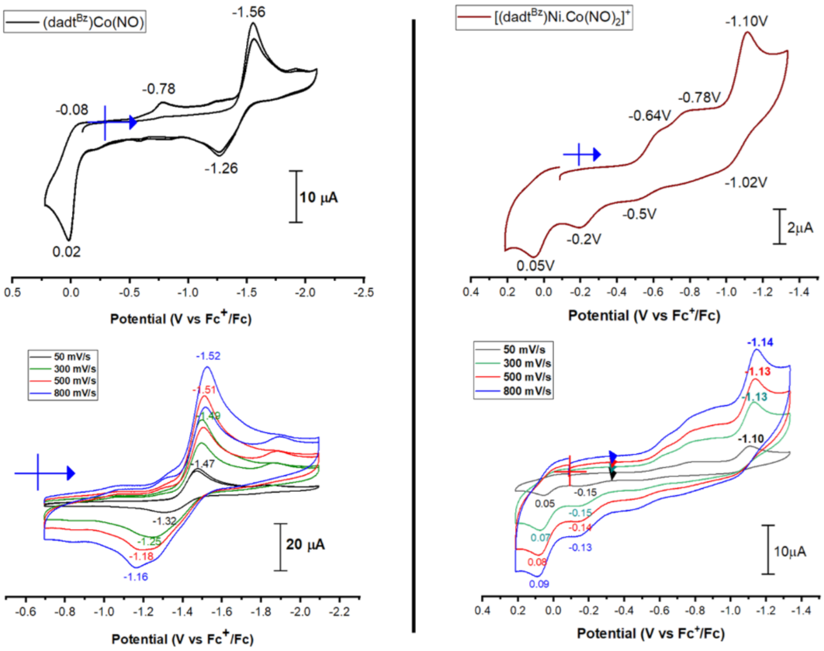

Figure S23: Cyclic voltammetry of (dadt^{Bz})Co(NO)(**A**) and [(dadt^{Bz})Ni•Co(NO)$_2$][PF$_6$] (**B**) in CH$_3$CN solution containing 0.1M [^{n}Bu$_4$N][PF$_6$] as the supporting electrolyte at room temperature under argon and referenced to Fc$^{+/0}$ ($E_{1/2}$= 0.0 V) as an internal standard.

A quasi-reversible reduction event at −1.56 V was observed for (dadt^{Bz})Co(NO) and assigned to the {Co(NO)}$^{8/9}$ couple. In contrast, the cyclic voltammogram of [(dadt^{Bz})NiCo(NO)$_2$](PF$_6$) gives one quasi-reversible reduction event at −1.10V assigned to the reduction of {Co(NO)$_2$}^{10} to {Co(NO)$_2$}^{11}. Similar reduction events were also observed for dinitrosyl cobalt complexes (DNCC) containing dppe (1,2-bis(diphenylphosphino)ethane), dppp (1,3-bis(diphenylphosphino)propane) and TMEDA (tetra-methyl-ethylenediamine) at a slightly negative reduction potential (−1.32 V, −1.21 V and −1.34 V, respectively); all are found to be reversible.[13]

S5. *Quantification of NO gas:*

Gas identification was accomplished with an Agilent Trace 1300 GC equipped with a thermal conductivity detector and a custom made 120 cm stainless steel column packed with Carbosieve-II from Sigma Aldrich. The carrier gas was Ar, and throughout the entire separation, the column was kept at 200 °C, while the detector was at 250 °C. Identification and quantification of NO produced during the reaction was accomplished by withdrawing 300 μL of the headspace using a 0.5 mL Valco Precision Sampling Syringe, Series A2 equipped with a Valco Precision Sampling syringe needle with a 5-point side port. N_2 is the first peak to elute from the column at 2.3 min, followed by NO at 2.6 min, and finally CH_4 at 4.15 min. Quantification of NO produced was determined by the relative response factor of NO and the internal standard, CH_4.

A calibration curve was generated by preparing vials containing known amounts of pure NO gas and 0.1 mL of CH_4 gas as reference (Figure S24). This was done by injecting varying amounts of NO in a stirring solution of MeCN (3 mL) followed by addition of 0.1 mL CH_4 in all the vials, capped with a rubber septum and sealed with parafilm. The vials were allowed to stir for 1 hr. Quantification of NO was accomplished by withdrawing 300 μL of the headspace using a gas-tight syringe and injecting into the GC. The calibration curve was generated by plotting the (area of NO/area of CH_4) (as obtained from the gas chromatograms) vs. (mL of NO/mL of CH_4 added).

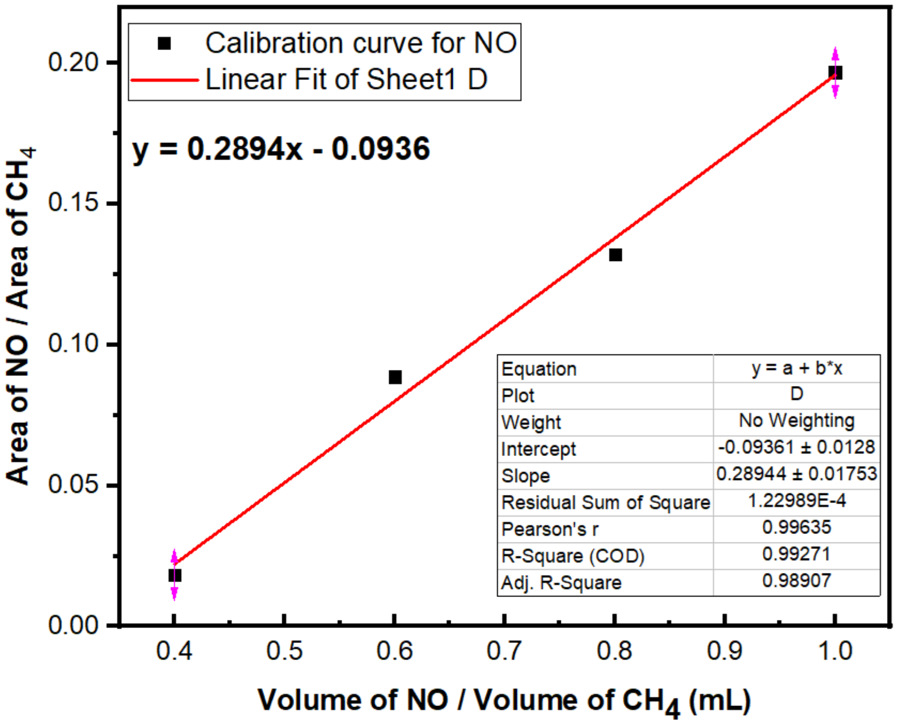

Figure S24: Calibration curve used for quantification of NO gas produced during the reaction.

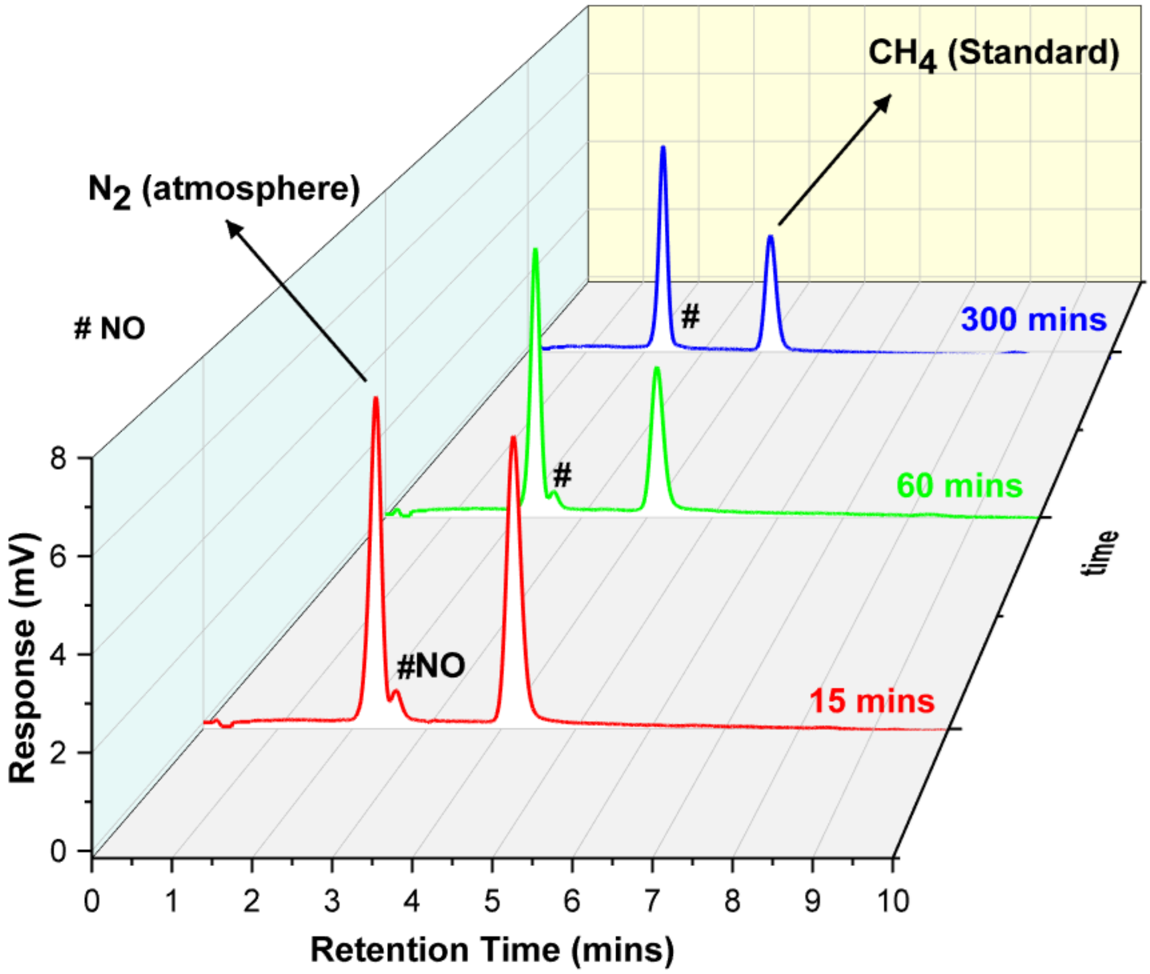

Gas	Retention Time
N_2	2.3 min
NO	2.56 min
CH_4	4.15 min

Figure S25: Plot of GC traces for the reaction of (dadt^{Bz})Co(NO) with $[Ni(NO)(CH_3NO_2)]^+$ in MeCN showing the presence of NO (#) gas, released during the reaction (in 15 mins (red trace), 60 mins (Green trace)). The released NO (#) gas is consumed over time (5 h (blue trace)). 0.1 mL of CH_4 was added as a standard at the beginning of the reaction.

Table S5: The area of NO and CH_4 as obtained from the GC at various time intervals.

	**Area of NO**	**Area of CH_4**	**Area of NO/ Area of CH_4**
After 15 mins	0.0388	1.3992	0.0277
After 60 mins	0.0275	0.7894	0.0348
After 5h	--	0.8921	--

The reaction was performed using 0.05 mmol of (dadt^{Bz})Co(NO) and 0.05 mmol of [Ni(NO)(CH_3NO_2)$_3$]$^+$. Based on the theoretical volume of NO that should be formed if all the Ni(NO) releases its NO (based on Proposed mechanism, Scheme 1) (100 % conversion = 0.05 mmol × 24.45 mL/mmol = 1.2225 mL (@ 1atm, 22 °C)), the volume of NO detected during the experiment at different time intervals and corresponding yields were given in the following Table S6.

Table S6: Yields of NO as determined from GC during the reaction at different time intervals.

	Area of NO / Area of CH_4	Volume of NO as obtained from Calibration curve	Yield
After 15 mins	0.0277	0.4191	34 %
After 60 mins	0.0348	0.4436	36 %
After 5 h	--	--	--

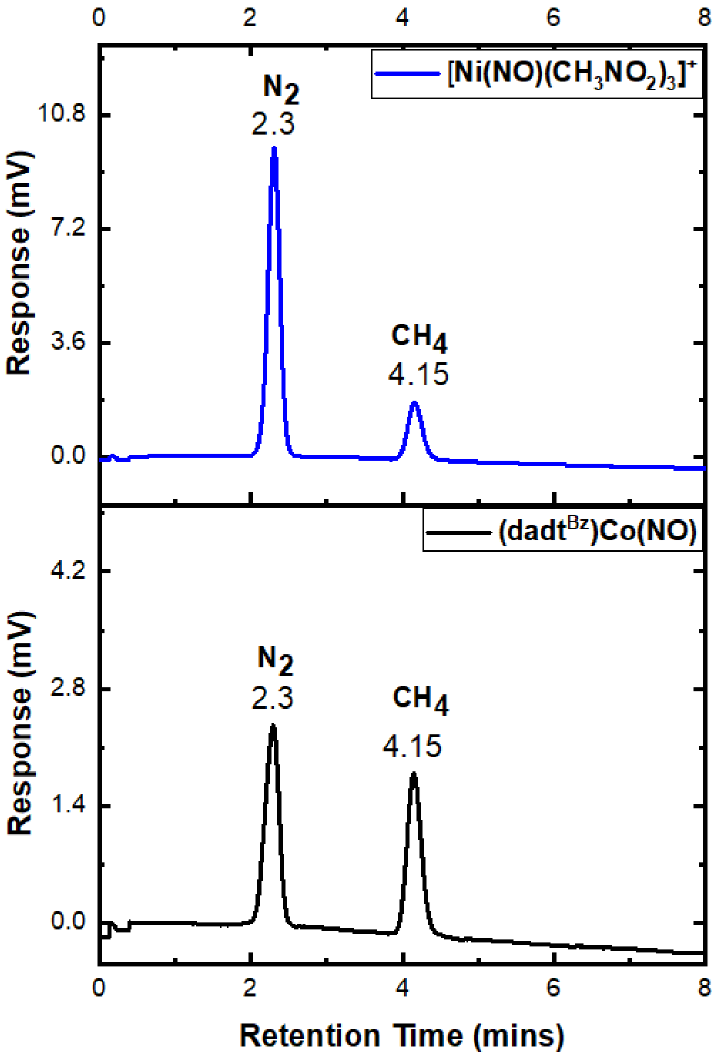

Figure S26: GC traces from headspace of 0.05 mM of (dadt^{Bz})Co(NO) (bottom) and 0.05 mM of [Ni(NO)(CH_3NO_2)$_3$]$^+$ (top) dissolved in 3mL MeCN solution showing no NO gas release after 1h. 0.1mL of CH_4 was added as internal standard.

S6. *DFT Calculation:*

DFT calculation was performed using the TPSSTPSS[14] functional, and the triple-ζ basis set 6-311++G(d,p)[15] in Gaussian16 Revision C.01.[16] This selection of functional and basis set was benchmarked against the ν(NO) stretches of the 2 isolated species (dadt^{Bz})Co(NO) and [(dadt^{Bz})Ni•Co(NO)$_2$]$^+$, Figure S27.

(A)

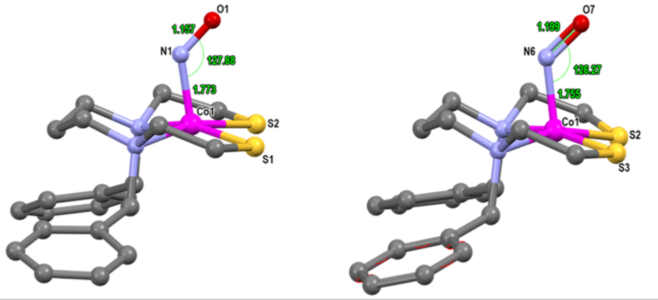

	Experimental	DFT Optimized
Co-N (Å)	1.773	1.755
N-O (Å)	1.157	1.199
∠Co-N-O (˚)	127.88	126.27
ν_{NO} (cm^{-1})	1603	1600

(B)

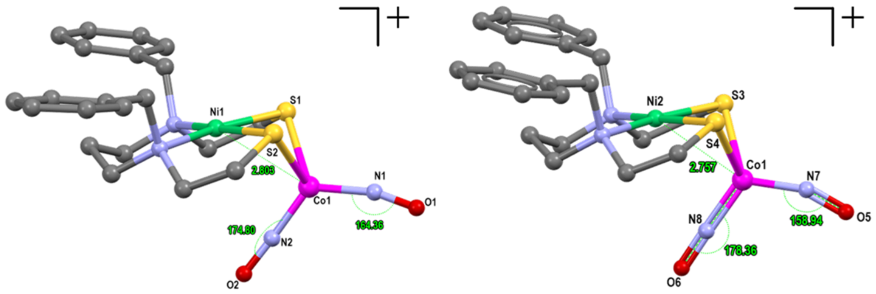

	Experimental	DFT Optimized
Ni-Co (Å)	2.803	2.757
∠Co-N-O (˚)	164.36, 174.80	158.94, 178.36
ν_{NO} (cm^{-1})	1780, 1842	1777, 1842

Figure S27: Experimental and DFT optimized structure of (dadt^{Bz})Co(NO) (**A**) and [(dadt^{Bz})Ni•Co(NO)$_2$]$^+$ (**B**) and their experimental and calculated IR stretching frequency (Scaling factor = 1.020).

The calculated free energy difference between the two isomers of the expected/hypothetical intermediate is given in Figure S28. The isomer having the NO and benzyl groups opposite to each other with respect to N_2S_2 plane (*trans* isomer) is found to be comparable in energy to the structure where the NO and the benzyl groups are on the same side (*cis* isomer) of the N_2S_2 plane. The calculated free energy difference (ΔG) between these two isomers is found to be only 1.03 kcal/mol in favour of the *trans* isomer. Electrostatic potential maps show no steric factors although the Co is found to be slightly more displaced from the mean N_2S_2 plane in the *cis* isomer (Figure S28).

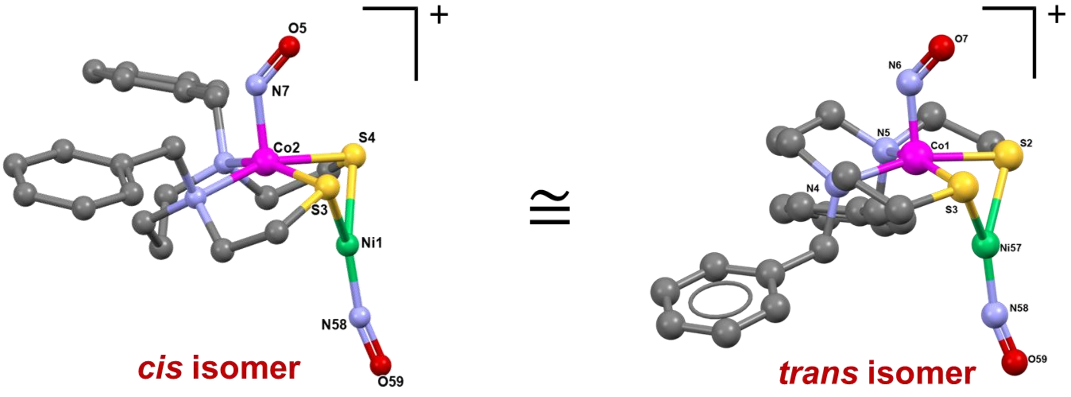

NO and benzyl groups are on the same side with respect to N_2S_2 plane	
Ni-Co	2.622 Å
∠Co-N-O	133.79˚
∠Ni-N-O	167.74˚
Co_{disp}-N_2S_2	0.341 Å

NO and benzyl groups are opposite to each other with respect to N_2S_2 plane	
Ni-Co	2.538 Å
∠Co-N-O	133.44˚
∠Ni-N-O	163.09˚
Co_{disp}-N_2S_2	0.272 Å

(ΔG) = 1.03 kcal/mol

Figure S28: Calculated energy difference between two isomers of the hypothetical intermediate.

The trans isomer described in Figure S28 is used to calculate the probable dinitrosyl heterobimetallic intermediates based on the reported complexes consisting of metallodithiolate as mono- or bidentate ligands.[17,18] All of the atoms present in the rearranged product are preserved in these intermediates, i.e., both the metal centers and NO ligands are present. MeCN solvent molecules were explicitly added to fill the coordination sphere of Ni in several cases. Relative free energy compared to the rearranged product and their respective calculated ν(NO) IR values are summarized in Figure S29. Based on the free energy of the DFT optimized structures, all of these heterobimetallic intermediates are found to be significantly higher in energy compared to the final product. The most probable intermediate is found to be the one with tri coordinated Ni(NO) species, $[(dadt^{Bz})Co(NO)•Ni(NO)]^+$ (lower energy), similar to $[(bipy)Ni(NO)]^+$ species. In case of $[(dadt^{Bz})Co(NO)•Ni(NO)(MeCN)]^+$, the calculated ν(NO) values were only found to be 12 cm^{-1} different (1640 and 1770 cm^{-1}) from the experimentally observed IR stretching frequency (1652 cm^{-1} and the other one masked by the product absorption band around 1780 cm^{-1}), while it is found to be significantly higher in energy compared to the isolated species (20.7 kcal/mol, Figure S29). IR stretching frequency of all other species were not observed or matched closely with the experimentally observed IR values. Although there are several possibilities, these calculations along with the observations of free NO gas in the headspace during the reaction suggests that the intermediate formed within time of mixing only contain one NO ligand.

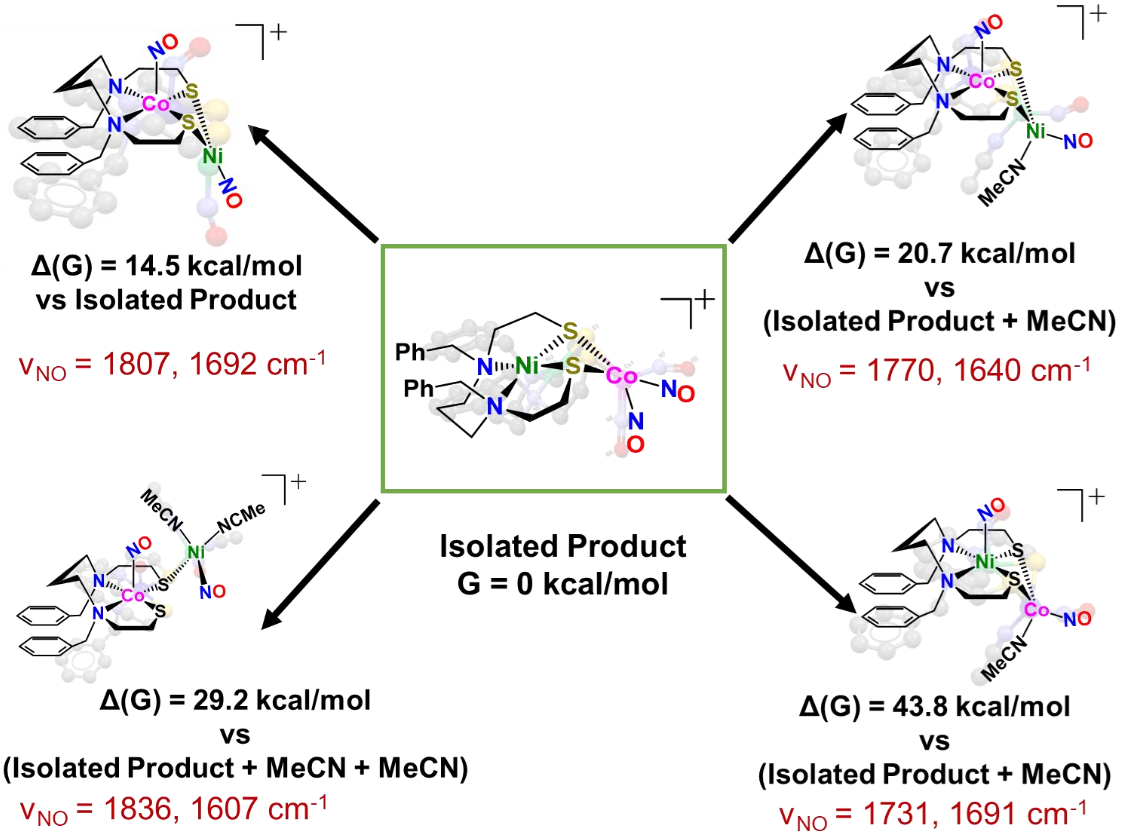

Figure S29: Relative free energy of the probable intermediates compared to the rearranged product and their respective calculated ν(NO) IR values.

S7. NO Transfer Literature:

The first report on NO transfer came from Armor *et al*. They have reported an acid-promoted metal dependent NO transfer from Co^{III} to Cr^{II} involving a labile aqua complex.[19] Further studies suggested that the transfer of the NO ligand occurs at highly acidic conditions due to axial NO ligand protonation.[20] Caulton and co-workers have studied the NO transfer reactivity from Co to various metal complexes of Fe, Ru, Rh, and Co, and proposed that the NO transfers readily to the coordinatively unsaturated metal complexes, either via a simple NO transfer or via halide exchange pathway.[21] Cook and co-workers, for the first time, reported a dissociative pathway in a NO transfer reaction from $Co(NO)(dmgH)_2$ to Hemoglobin (Hb).[22] Kadish *et al.* speculated the effect of metal-center on a NO transfer reaction.[23] They have proposed an intermediate species formation (associative mechanism) before NO transfer. Lippard and co-workers also suggested a dissociative NO transfer pathway from Mn to Fe bearing a tropocarnado ligand.[24] A *μ*-NO bridge intermediate was discussed in a NO transfer reaction of Ru to Fe-center of Hb and myoglobin (Mb).[25] Nam and co-workers reported NO transfer reactivity of cobalt complex induced by ligand size and the spin-state of the acceptor/donor metal complexes.[26] Recently, Kumar and co-workers have shown that NO transfer from a Co^{III} complex to Cr^{II} occurs via a bridging NO intermediate.[27] All these reports suggest that an extensive amount of work has been done on NO transfer for over five decades. A simple NO transfer has been observed between two metals in separate complexes previously, but in our case the NO transfer is within a heterobimetallic discrete molecule. This is a fundamental discovery.

S8. Supplementary References:

[1] A. M. Wright, G. Wu, T. W. Hayton, *Inorg. Chem.* **2011**, *50*, 11746–11753.

[2] Bruker (**2019**). *APEX3* (v2019.1-0) Bruker AXS Inc., Madison, Wisconsin, USA.

[3] G. M. Sheldrick, (**2016**). *SADABS.* University of Göttingen, Germany.

[4] G. M. Sheldrick, *Acta Crystallographica Section A* **2015,** *71* (1), 3-8.

[5] G. M. Sheldrick, *Acta Crystallographica Section C* **2015,** *71* (1), 3-8.

[6] O. V. Dolomanov, L. J. Bourhis, R. J. Gildea, J. A. K. Howard, H. Puschmann, OLEX2: a complete structure solution, refinement, and analysis program. *Journal of Applied Crystallography* **2009,** *42* (2), 339-341.

[7] C. F. Macrae, I. Sovago, S. J. Cottrell, P. T. A. Galek, P. McCabe, E. Pidcock, M. Platings, G. P. Shields, J. S. Stevens, M. Towler and P. A. Wood*, J. Appl. Cryst.,* **53**, *226-235*, 2020.

[8] H. Kurihara, A. Ohta, K. Fujisawa, *Inorganics* **2019**, *7*, 116.

[9] J.-L. Roustan, N. Ansari, Y. L. Page, J.-P. Charland, *Can. J. Chem.* **1992**, *70*, 1650–1657.

[10] (a) K. Fujisawa, S. Soma, H. Kurihara, H. T. Dong, M. Bilodeau, N. Lehnert, *Dalton Transactions* **2017**, *46*, 13273–13289. (b) M. R. Crimmin, L. E. Rosebrugh, N. C. Tomson, T. Weyhermüller, R. G. Bergman, F. D. Toste, K. Wieghardt, *Journal of Organometallic Chemistry* **2011**, *696*, 3974–3981.

[11] J. A. Kaduk, J. A. Ibers, *Inorg. Chem.* **1977**, *16*, 3283–3287.

[12] B. E. Reichert, *Acta Cryst B* **1976**, *32*, 1934–1936.

[13] T. Zhao, Y. Zhang, P. Wang, S. Li, Z. Yang, M. Yang, *Inorganic Chemistry Communications* **2023**, *149*, 110418.

[14] J. Tao, J. P. Perdew, V. N. Staroverov, G. E. Scuseria, *Phys. Rev. Lett.* **2003**, *91*, 146401.

[15] R. Krishnan, J. S. Binkley, R. Seeger, J. A. Pople, *The Journal of Chemical Physics* **2008**, *72*, 650–654.

[16] M. J. Frisch, G. W. Trucks, H. B. Schlegel, G. E. Scuseria, M. A. Robb, J. R. Cheeseman, G. Scalmani, V. Barone, G. A. Petersson, H. Nakatsuji, X. Li, M. Caricato, A. V. Marenich, J. Bloino, B. G. Janesko, R. Gomperts, B. Mennucci, H. P. Hratchian, J. V. Ortiz, A. F. Izmaylov, J. L. Sonnenberg, Williams, F. Ding, F. Lipparini, F. Egidi, J. Goings, B. Peng, A. Petrone, T. Henderson, D. Ranasinghe, V. G. Zakrzewski, J. Gao, N. Rega, G. Zheng, W. Liang, M. Hada, M. Ehara, K. Toyota, R. Fukuda, J. Hasegawa, M. Ishida, T. Nakajima, Y. Honda, O. Kitao, H. Nakai, T. Vreven, K. Throssell, J. A. Montgomery Jr., J. E. Peralta, F. Ogliaro, M. J. Bearpark, J. J. Heyd, E. N. Brothers, K. N. Kudin, V. N. Staroverov, T. A. Keith, R. Kobayashi, J. Normand, K. Raghavachari, A. P. Rendell, J. C. Burant, S. S. Iyengar, J. Tomasi, M. Cossi, J. M. Millam, M. Klene, C. Adamo, R. Cammi, J. W. Ochterski, R. L. Martin, K. Morokuma, O. Farkas, J. B. Foresman and D. J. Fox, Gaussian Inc., Wallingford, CT, 2016.

[17] J. A. Denny, M. Y. Darensbourg, *Chem. Rev.* **2015**, *115*, 5248–5273.

[18] J. A. Denny, M. Y. Darensbourg, *Coordination Chemistry Reviews* **2016**, *324*, 82–89.

[19] J. Armor, *Inorganic Chemistry* **1973**, *12*, 1959–1961.

[20] R. L. Roberts, D. W. Carlyle, G. L. Blackmer, *Inorganic Chemistry* **1975**, *14*, 2739–2744.

[21] C. B. Ungermann, K. G. Caulton, *Journal of the American Chemical Society* **1976**, *98*, 3862–3868.

[22] M. P. Doyle, R. A. Pickering, R. L. Dykstra, B. R. Cook, *Journal of the American Chemical Society* **1982**, *104*, 3392–3397.

[23] X. H. Mu, K. M. Kadish, *Inorganic Chemistry* **1990**, *29*, 1031–1036.

[24] K. J. Franz, S. J. Lippard, *Inorganic Chemistry* **2000**, *39*, 3722–3723.

[25] G. Metzker, P. P. Lopes, A. C. da Silva, S. C. da Silva, D. W. Franco, *Inorganic Chemistry* **2014**, *53*, 4475–4481.

[26] P. Kumar, Y.-M. Lee, L. Hu, J. Chen, Y. J. Park, J. Yao, H. Chen, K. D. Karlin, W. Nam, *Journal of the American Chemical Society* **2016**, *138*, 7753–7762.

[27] S. Das, S. Ray, T. Devi, S. Ghosh, S. S. Harmalkar, S. N. Dhuri, P. Mondal, P. Kumar, *Chemical Science* **2022**, *13*, 1706–1714.
